# Associations between novel triglyceride-glucose-related indices and the incidence of hypertension among Chinese middle-aged and elderly adults: a nationwide prospective cohort study

**DOI:** 10.1186/s40842-025-00255-3

**Published:** 2025-12-29

**Authors:** Zhoubin Cao, Jian Wang, Naidong Pang, Yingxiang Lin, Xingpeng Liu

**Affiliations:** 1 Department of Cardiology, Heart Center, Beijing Chao-Yang Hospital, Capital Medical University, Beijing, China; 2 Department of Respiratory and Critical Care Medicine, Beijing Institute of Respiratory Medicine and Beijing Chao-Yang Hospital, Capital Medical University, Beijing, China; 3 Department of Cardiology, HTRM Cardiovascular Hospital, Dezhou, Shandong, China

**Keywords:** Triglyceride-glucose-related indices, Hypertension risk, China health and retirement longitudinal study

## Abstract

**Background:**

Novel triglyceride-glucose-related(TyG-related) indices have garnered increasing interest as predictors of cardiometabolic risk. However, their prospective associations with the incidence of hypertension among middle-aged and elderly adults are insufficiently characterized.

**Methods:**

We analysed 4,541 participants aged 45 years or older, with no history of hypertension at baseline, from the China Health and Retirement Longitudinal Study (CHARLS). Cox proportional hazards regression and restricted cubic spline (RCS) regression analyses were applied to assess the associations between the novel TyG-related indices and incident hypertension. Time-dependent ROC curves were used to assess the predictive discriminatory ability of the model at different time points over time. We used the NRI and IDI to evaluate the improvement in risk prediction capability of TyG-related indices compared to the TyG index alone and BMI. The robustness of the study findings was examined, to some extent, through subgroup and sensitivity analyses.

**Results:**

In the longitudinal analysis, 1,484 participants (32.7%) experienced an new-onset hypertension. In fully adjusted models, each 1-SD increase in TyG–BRI, TyG–ABSI, TyG–WWI, and TyG–CVAI was associated with hypertension hazards of 1.21 (95% CI, 1.14–1.28), 1.09 (1.03–1.16), 1.12 (1.05–1.19), and 1.21 (1.18–1.28), respectively. RCS analysis revealed a significant positive linear association between TyG-related indices and the risk of incident hypertension. The time-dependent ROC curves demonstrated that TyG-BRI and TyG-CVAI consistently exhibited higher AUC values over time compared with TyG-ABSI and TyG-WWI, indicating better discriminatory performance, although the Delong test suggested that these differences were not statistically significant in most cases. TyG-BRI and TyG-CVAI demonstrated better predictive performance compared to the TyG index and base model, as evidenced by higher improvements in both NRI and IDI across multiple follow-up periods. The findings were consistent across multiple subgroup and sensitivity analyses.

**Conclusion:**

All four TyG-related indices were significantly associated with the risk of incident hypertension among middle-aged and older Chinese adults, emphasizing the need to increase attention to these indices to enhance hypertension detection and prevention in this population.

**Supplementary Information:**

The online version contains supplementary material available at 10.1186/s40842-025-00255-3.

## Introduction

Hypertension is a major global public health challenge and a leading risk factor for cardiovascular disease and premature death worldwide, presenting significant public health challenges and imposing an economic burden on patients [1]. In China, the prevalence of hypertension has increased substantially over the past few decades, currently affecting approximately 23.2% of the adult population [2]. The prevention of hypertension has become an urgent priority in public health.

Insulin resistance (IR), characterized by impaired glucose metabolism in skeletal muscle, adipose tissue, and the liver, plays a pivotal role in the development of cardiovascular diseases and hypertension [3, 4]. Although the hyperinsulinemic -euglycemic clamp (HEC) remains the gold standard for assessing IR, its technical complexity and high cost limit its practicality in routine clinical settings [5]. This underscores the need for a reliable, cost-effective, and easily applicable alternative for widespread IR evaluation. In this context, simplified indices such as the triglyceride-glucose (TyG) index—which has been proven to be a highly effective surrogate marker for IR—offer significant value for broader clinical implementation [6, 7].

Although the TyG index is sensitive to insulin resistance, it may underestimate metabolic risk in individuals with marked obesity but normal triglyceride and glucose levels. Several studies have suggested that the combination of the TyG index and obesity-related indices is associated with an increased risk of hypertension, as it more effectively reflects both insulin resistance and the interplay between obesity and hypertension [8–10]. Nevertheless, the associations between TyG-related indices and hypertension remain uncertain. In this study, we applied Cox proportional hazards models and restricted cubic spline analyses to examine the relationships between TyG-related indices and incident hypertension among middle-aged and older Chinese adults, with the aim of offering new insights for hypertension prevention and management in this population.

## Materials and methods

### Study design and population

The data for the present investigation were obtained from the CHARLS, a nationwide prospective cohort study. CHARLS employs a multistage stratified probability proportional-to-size sampling approach to recruit participants from both rural and urban regions covering 150 counties or districts across 28 provinces in China. Five regular surveys were conducted between 2011 and 2020. Detailed descriptions of the study design and cohort profile have been previously reported in the literature [11]. The CHARLS study was conducted in full compliance with the principles of the Declaration of Helsinki and received ethical approval from the Institutional Review Board of Peking University (IRB0000105211015).

**Fig. 1:**
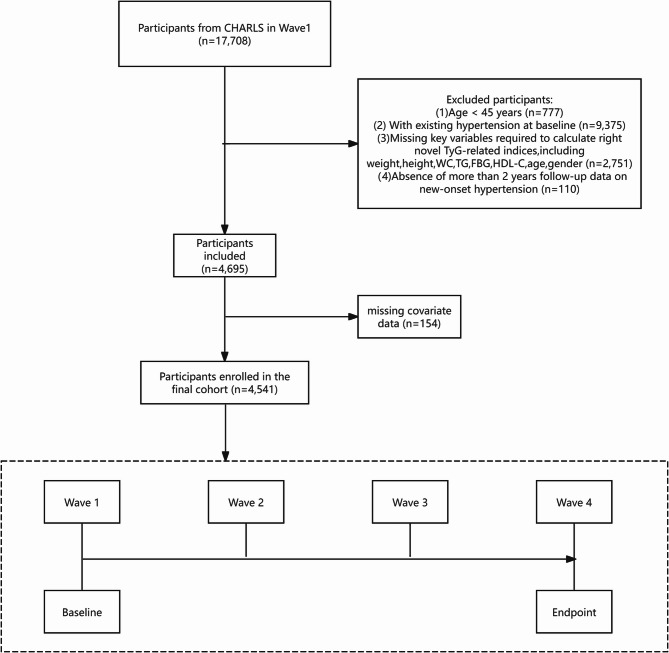
Flowchart of participant selection

For our analysis, we selected data from the 2011, 2013, 2015, and 2018 waves. A total of 17,708 individuals were initially enrolled in the baseline cohort. The following exclusion criteria were applied to construct the final analytic sample: (1) age less than 45 years at baseline; (2) existing hypertension at baseline or missing data to diagnose hypertension; (3) unavailable data or missing key variables to calculate novel TyG-related indices; (4) absence of more than 2 years follow-up data on new-onset hypertension; (5) missing covariate data. After exclusion, a total of 4,541 participants were eligible for inclusion in the final cohort. A schematic diagram detailing the selection process is presented in Fig. 1.

### Data assessment and definitions

#### Definitions of four TyG-related indices

Fasting venous blood samples were collected from participants after an at least 8-hour fast and were then transported to Beijing for analysis. Triglyceride (TG) and fasting plasma glucose (FPG) levels were measured using enzymatic colorimetric methods at the Clinical Laboratory of Capital Medical University, which is accredited by the Beijing Health Bureau. The coefficient of variation for both TG and FPG measurements was less than 2%. Triglyceride-glucose index–body roundness index(TyG-BRI), triglyceride-glucose index–a body shape index(TyG-ABSI), triglyceride-glucose index–weight-adjusted waist index(TyG-WWI) and triglyceride-glucose index–Chinese visceral adiposity index(TyG-CVAI) are novel indices integrating the TyG index and anthropometric parameters, designed to enhance the evaluation of metabolic dysfunction and visceral adiposity. The TyG index is the natural logarithm of fasting triglycerides multiplied by fasting glucose, divided by two. The indices for Body Roundness (BRI), A Body Shape (ABSI), and Weight-Waist (WWI) are computed utilizing fundamental anthropometric data, namely waist circumference, stature, and body mass, to assess body composition. CVAI is calculated using age and waist circumference, in addition to BMI, triglycerides, and HDL levels. The calculation of TyG indices involves the following formula [12–15]:

$$\:TyG\:-\:BRI\:=\:TyG\:\times\:\:BRI$$

$$\:TyG\:-\:ABSI\:=\:TyG\:\times\:\:ABSI$$

$$\:TyG\:-\:WWI\:=\:TyG\:\times\:\:WWI$$

$$\:TyG\:-\:CVAI\:=\:TyG\:\times\:\:CVAI$$

$$\:TyG\:=\:ln\:\left[TG\right(\:mg/dL)\:\times\:\:FBG(mg/dL)/2]$$

$$\:BRI=364.2-365.5\times\:\sqrt{1-{\left(WC\left(cm\right)/2\pi\:\right)}^{2}/{\left(Height\left(cm\right)/2\right)}^{2}}$$

$$\:ABSI\:=\:WC\:\left(m\right)\:/{\left(BMI\right(kg/{m}^{2})}^{\frac{2}{3}}\:\times\:\:{Height\:\left(m\right)}^{\frac{1}{2}})\:$$

$$\:W\:W\:I\:=\:WC\:\left(cm\right)\:/\sqrt{Weight\:\left(kg\right)}$$

$$\begin{aligned}\:CVAI\:=\:&-267.93\:+\:0.68\:\times\:\:age\:\left(years\right)\:\\&+\:0.03\:\times\:\:BMI\:(kg/{m}^{2})\:\\&+\:4.00\:\times\:\:WC\:\left(cm\right)\:\\&+\:22.00\:\times\:\:{log}_{10}\left(TG\right)\:(mmol/L)\:\\&-\:16.32\:\times\:\:HDL\:\\&-\:C\:(mmol/L)\:in\:male\end{aligned}$$

$$\begin{aligned}\:CVAI\:=\:&-187.32\:+\:1.71\:\times\:\:age\:\left(years\right)\:\\&+\:4.23\:\times\:\:BMI\:(kg/{m}^{2})\:\\&+\:1.12\:\times\:\:WC\:\left(cm\right)\:\\&+\:39.76\:\times\:\:{log}_{10}\:\left(TG\right)\:(mmol/L)\:\\&-\:11.66\:\times\:\:HDL\:\\&-\:C\:(mmol/L)\:in\:female\end{aligned}$$

### Definition of hypertension

After participants rested in a seated position for at least 5 min, blood pressure was assessed three times at 5-minute intervals using an electronic monitor (Omron HEM-7200). The mean of these three readings was considered for analysis. Incident hypertension was defined as newly diagnosed hypertension during the follow-up period. Hypertension was defined as systolic blood pressure (SBP) ≥ 140 mmHg, diastolic blood pressure (DBP) ≥ 90 mmHg, a diagnosis of hypertension by a doctor, or current use of antihypertensive medication [16]. The date of onset was considered the date of that follow-up visit.

### Covariates

In line with the Chinese hypertension guidelines and previous studies on hypertension in China, we included covariates such as sex, age, education level (below middle school, middle school and high school, or college or higher), marital status (married or other), residence (rural or urban), smoking status (now, former and never), alcohol consumption (now, former or never), and history of dyslipidemia, diabetes, heart disease, stroke, and kidney disease. The laboratory examination included tests for glycosylated hemoglobin type A1c (HbA1c), total cholesterol (TC), and uric acid(UA). The estimated glomerular filtration rate (eGFR) was calculated via the Chronic Kidney Disease Epidemiology Collaboration (CKD-EPI) 2009 formula [17]. Heart disease was defined by either a diagnosis from a doctor or the administration of treatment. Stroke was defined as being diagnosed by a doctor or receiving treatment for stroke. Dyslipidemia is characterized by a TC level of ≥ 240 mg/dL, a TG level of ≥ 150 mg/dL, an LDL-C level of ≥ 160 mg/dL, an HDL-C level of < 40 mg/dL, or ongoing lipid-lowering treatments, or a medical diagnosis [18]. Diabetes was defined as an FBG level ≥ 126 mg/dL, or an HbA1c level ≥ 6.5%, or antidiabetic medication usage, or a medical diagnosis [19]. Kidney disease was defined as an eGFR < 60 ml/min/1.73m^2^, receiving treatment for kidney issues, or being diagnosed by a doctor [20]. Lung disease was defined by either a diagnosis from a doctor or the administration of treatment.Asthma was defined by either a diagnosis from a doctor.

### Handling of missing data and data preprocessing

Most variables had relatively low proportions of missing data. The missing rates were as follows: diabetes (1.5%), dyslipidemia (0.9%), HbA1c (0.9%), cardiovascular disease (0.4%), lung disease (0.4%), asthma (0.3%), smoking (0.2%), stroke (0.2%), drinking (< 0.1%), eGFR (< 0.1%). We performed the primary analysis using the complete case data. Additionally, a sensitivity analysis with multiple imputation for missing covariates was conducted to address potential selection bias.

A percentile-based Winsorization procedure was implemented to reduce the impact of outliers and strengthen the robustness of the analysis. For the variables TyG-BRI, TyG-ABSI, TyG-WWI, and TyG-CVAI, the extreme 1% of values at each tail of the distribution (below the 1st and above the 99th percentiles) were replaced with the values at these percentile thresholds. This processing step was performed prior to any statistical modeling.

### Statistical analysis

The baseline characteristics of the study participants, categorized by the quartiles of TyG-related indices, for continuous variables that displayed a normal distribution, statistics are presented as the means(standard deviation(SD)). For continuous variables that did not follow a normal distribution, the median and interquartile ranges were utilized for statistical description. Differences between groups were assessed with the χ² test for categorical variables and the Kruskal–Wallis or Mann–Whitney tests for continuous variables. Spearman correlation coefficients were calculated to evaluate the interrelationships between the TyG index and the four TyG-related indices.

Survival curves were generated via the Kaplan-Meier (KM) method to estimate the cumulative incidence of hypertension events over time. The participants were stratified by TyG-related indices. The log-rank test was used to compare survival distributions between groups.

To evaluate the relationships between the TyG-related indices and hypertension incidence, three Cox proportional hazards models were established, and hazard ratios (HRs) with their 95% confidence intervals (95% CIs) are presented. Model 1 was unadjusted. In Model 2, adjustments for age, gender, smoking status, drinking status, BMI, residence, eGFR, education level, marital status, TC, UA, and HbA1c were included. In Model 3, adjustments for all covariates included in Model 2, in addition to heart disease, dyslipidemia, diabetes, stroke, kidney disease, lung disease, and asthma.In the multicollinearity test, the variance inflation factor (VIF) [21] for each variable included in our analysis was determined, which was below 5 (Additional file 2: Table S1), suggesting no evidence of significant multicollinearity. To enhance comparability across models, the four TyG-related indices were standardized to z scores, allowing effect estimates to represent the change in hypertension risk per SD increase in each index. We constructed calibration plots for the fully adjusted model based on the Kaplan–Meier method and evaluated the calibration of the Cox proportional hazards model using the Grønnesby–Borgan test. The accuracy of the prediction model was assessed using the Brier score curve. The proportional hazards assumption for the Cox regression model was assessed using the Cox-ZPH test (Additional file 2: Figure S1-S3, Table S2).

A multivariable adjusted (fully adjusted) RCS model analysis was fitted within the Cox proportional hazards framework, to examine the linearity and the dose-reponse relationship between the TyG-related indices and hypertension incidence. Four knots were placed at the 5th, 35th, 65th, and 95th percentiles of TyG-related indices, using the median as the reference point. In the presence of detected nonlinearity, a threshold effect analysis was performed on the Cox regression model. The breakpoint was identified, and a likelihood ratio test was conducted to compare the fit of the linear model with that of the segmented model, allowing for separate estimation of hazard associations below and above the threshold.

To further evaluate the discriminatory capacity of different metabolic indices for predicting incident hypertension, time-dependent ROC curve analyses were performed based on the fully adjusted Cox proportional hazards models (Model 3).The area under the curve (AUC) was calculated for each index to compare their predictive performance. Analyses were conducted at 24, 48, and 60 months, as well as at the median follow-up time, taking into account the follow-up duration of the database and clinical relevance.Pairwise comparisons based on DeLong’s test were conducted to identify indices with superior predictive performance, followed by an assessment of the incremental predictive value using the Integrated Discrimination Improvement (IDI) index and Net Reclassification Index (NRI).

Furthermore, to detect potential modifications, a variety of subgroup analyses and interaction analyses were performed. The participants were stratified into diverse subgroups, according to age (< 60, ≥ 60 years), gender, smoking status, drinking status, BMI(< 24,24–28, ≥ 28), the presence of dyslipidemia, kidney disease and diabetes. To control the false positive risk due to multiple comparisons, the Bonferroni correction was applied to the initial significance level of the subgroup analyses.

Finally, the study results were further validated through a set of sensitivity analyses. First, we reanalyzed the data after excluding participants who already had hypertension at Wave 2 (2013) to test whether the relatively short-term onset of hypertension had an effect on the primary outcome. Second, a sensitivity analysis was performed to evaluate the influence of extreme values by re-running the Cox regression models with the original TyG-related indices before any Winsorization was applied. Third, to minimize potential bias, we conducted Cox regression analyses using the multiply imputed datasets. Finally, E-values were computed to quantify the minimum strength of association that an unmeasured confounder would need to have with both the exposure and the outcome to explain away the observed associations.

All the statistical analyses were performed with R software version 4.4.3 (http://www.R-project.org/). A two-tailed *p* value < 0.05 was considered statistically significant.

## Results

### Baseline characteristics of the participants

Among the 4,541 participants (2,151 men and 2,390 women; median age 56 years), those who developed new-onset hypertension (*n* = 1,484) differed markedly at baseline from those who did not (Table 1). Compared with non-hypertension participants, patients had higher median values of age, BMI, SBP, DBP, FBG, TG, UA, LDL-C, HbA1c, TC, the TyG index, and all TyG-related indices (BRI, TyG-BRI, ABSI, TyG-ABSI, WWI, TyG-WWI, CVAI, and TyG-CVAI) (all  *p* value< 0.05). In contrast, eGFR was lower among hypertension patients (p < 0.05). No significant differences were observed in HDL-C, BUN, and Scr among the groups.

**Table 1:** Baseline characteristics of the participants

	ALL	Non hypertension	Hypertension	p.overall
	*N* = 4541	*N* = 3057	*N* = 1484	
Age, years	56.00 [50.00;63.00]	56.00 [49.00;61.00]	59.00 [53.00;66.00]	<0.001
Gender, n%				0.153
Male	2151 (47.37%)	1425 (46.61%)	726 (48.92%)	
Female	2390 (52.63%)	1632 (53.39%)	758 (51.08%)	
Marry, n%				0.002
Yes	3923 (86.39%)	2675 (87.50%)	1248 (84.10%)	
Education, n%				0.005
Below middle school	3071 (67.63%)	2019 (66.05%)	1052 (70.89%)	
Middle school and high school	1419 (31.25%)	1001 (32.74%)	418 (28.17%)	
College or higher	51 (1.12%)	37 (1.21%)	14 (0.94%)	
Residence, n%				0.403
Urban	1530 (33.69%)	1043 (34.12%)	487 (32.82%)	
Rural	3011 (66.31%)	2014 (65.88%)	997 (67.18%)	
BMI (kg/m2)	22.53 [20.41;24.82]	22.37 [20.32;24.58]	22.92 [20.65;25.46]	<0.001
Drinking, n%				0.155
Never	2652 (58.40%)	1815 (59.37%)	837 (56.40%)	
Current	1575 (34.68%)	1038 (33.95%)	537 (36.19%)	
Former	314 (6.91%)	204 (6.67%)	110 (7.41%)	
Smoking, n%				0.367
Never	2741 (60.36%)	1866 (61.04%)	875 (58.96%)	
Now	1444 (31.80%)	959 (31.37%)	485 (32.68%)	
Former	356 (7.84%)	232 (7.59%)	124 (8.36%)	
Diabetes, n%:				<0.001
No	3990 (87.87%)	2727 (89.21%)	1263 (85.11%)	
Yes	551 (12.13%)	330 (10.79%)	221 (14.89%)	
Dyslipidemia, n%:				<0.001
No	2614 (57.56%)	1827 (59.76%)	787 (53.03%)	
Yes	1927 (42.44%)	1230 (40.24%)	697 (46.97%)	
Heart disease, n%:				0.083
No	4117 (90.66%)	2788 (91.20%)	1329 (89.56%)	
Yes	424 (9.34%)	269 (8.80%)	155 (10.44%)	
Kidney disease, n%				0.104
No	4152 (91.43%)	2810 (91.92%)	1342 (90.43%)	
Yes	389 (8.57%)	247 (8.08%)	142 (9.57%)	
Stroke, n%				0.014
No	4481 (98.68%)	3026 (98.99%)	1455 (98.05%)	
Yes	60 (1.32%)	31 (1.01%)	29 (1.95%)	
Lung disease, n%				0.026
No	4032 (88.79%)	2737 (89.53%)	1295 (87.26%)	
Yes	509 (11.21%)	320 (10.47%)	189 (12.74%)	
Asthma, n%				0.001
No	4343 (95.64%)	2946 (96.37%)	1397 (94.14%)	
Yes	198 (4.36%)	111 (3.63%)	87 (5.86%)	
SBP(mmHg)	118.33 [109.67;127.33]	115.67 [108.00;124.00]	124.67 [116.00;132.33]	<0.001
DBP(mmHg)	70.33 [64.33;76.67]	69.33 [63.33;75.33]	73.33 [66.67;79.00]	<0.001
eGFR	96.56 [86.90;104.03]	97.34 [87.72;104.30]	95.06 [85.53;102.78]	<0.001
FBG (mg/dl)	100.80 [93.42;109.98]	99.72 [92.88;108.72]	102.60 [94.86;112.32]	<0.001
TG (mg/dl)	96.46 [70.80;139.83]	94.69 [69.03;135.40]	100.00 [74.34;147.79]	<0.001
Scr (mg/dl)	0.75 [0.64;0.87]	0.75 [0.64;0.86]	0.75 [0.64;0.88]	0.528
UA (mg/dl)	4.17 [3.49;4.95]	4.13 [3.47;4.89]	4.24 [3.52;5.10]	0.001
BUN (mg/dl)	15.07 [12.49;18.07]	15.04 [12.55;17.95]	15.10 [12.44;18.26]	0.762
LDL-C (mg/dl)	114.05 [93.17;135.70]	113.27 [92.78;134.92]	115.21 [93.94;138.40]	0.046
HDL-C (mg/dl)	51.03 [41.75;61.47]	51.03 [42.14;61.08]	51.03 [40.59;61.47]	0.551
HbA1c (%)	5.10 [4.90;5.40]	5.10 [4.90;5.40]	5.20 [4.90;5.50]	<0.001
TC (mg/dl)	188.66 [165.85;213.40]	187.11 [165.08;210.70]	190.98 [167.78;217.66]	<0.001
TyG	8.50 [8.15;8.91]	8.47 [8.13;8.87]	8.55 [8.23;8.99]	<0.001
BRI	3.74 [3.01;4.67]	3.64 [2.94;4.53]	3.96 [3.18;4.96]	<0.001
TyG-BRI	31.87 [25.11;40.61]	30.84 [24.43;39.23]	33.89 [26.73;43.28]	<0.001
ABSI	0.08 [0.08;0.09]	0.08 [0.08;0.09]	0.08 [0.08;0.09]	<0.001
TyG-ABSI	0.70 [0.66;0.75]	0.69 [0.65;0.74]	0.71 [0.67;0.76]	<0.001
WWI	10.98 [10.45;11.54]	10.92 [10.40;11.47]	11.10 [10.56;11.68]	<0.001
TyG-WWI	93.62 [86.88;100.95]	92.85 [85.93;100.14]	95.15 [88.93;103.05]	<0.001
CVAI	83.28 [60.09;110.18]	79.41 [57.55;103.73]	92.41 [65.73;119.42]	<0.001
TyG-CVAI	706.41 [497.80;962.03]	674.79 [472.16;907.06]	795.12 [550.08;1055.98]	<0.001

Additionally, hypertension patients had a significantly greater incidence of dyslipidemia, stroke, lung disease, asthma and diabetes, with all differences reaching statistical significance ( *p* value< 0.05). The participants were divided into quartiles (Q1–Q4) for TyG-related indices (TyG-BRI, TyG-ABSI, TyG-WWI, and TyG-CVAI). Baseline characteristics across these quartiles are presented in Additional file 1 (Additional file 1 Table S1-S4).

Spearman correlation analysis revealed moderate associations between the TyG index and TyG-related indices, with coefficients ranging from 0.45 to 0.71. The TyG-ABSI correlated most strongly with TyG index (*r* = 0.71), followed by the TyG-WWI (*r* = 0.70), TyG-CVAI (*r* = 0.59), and TyG-BRI (*r* = 0.45).

### Relationship between TyG-related indices and the risk of new-onset hypertension

**Fig. 2:**
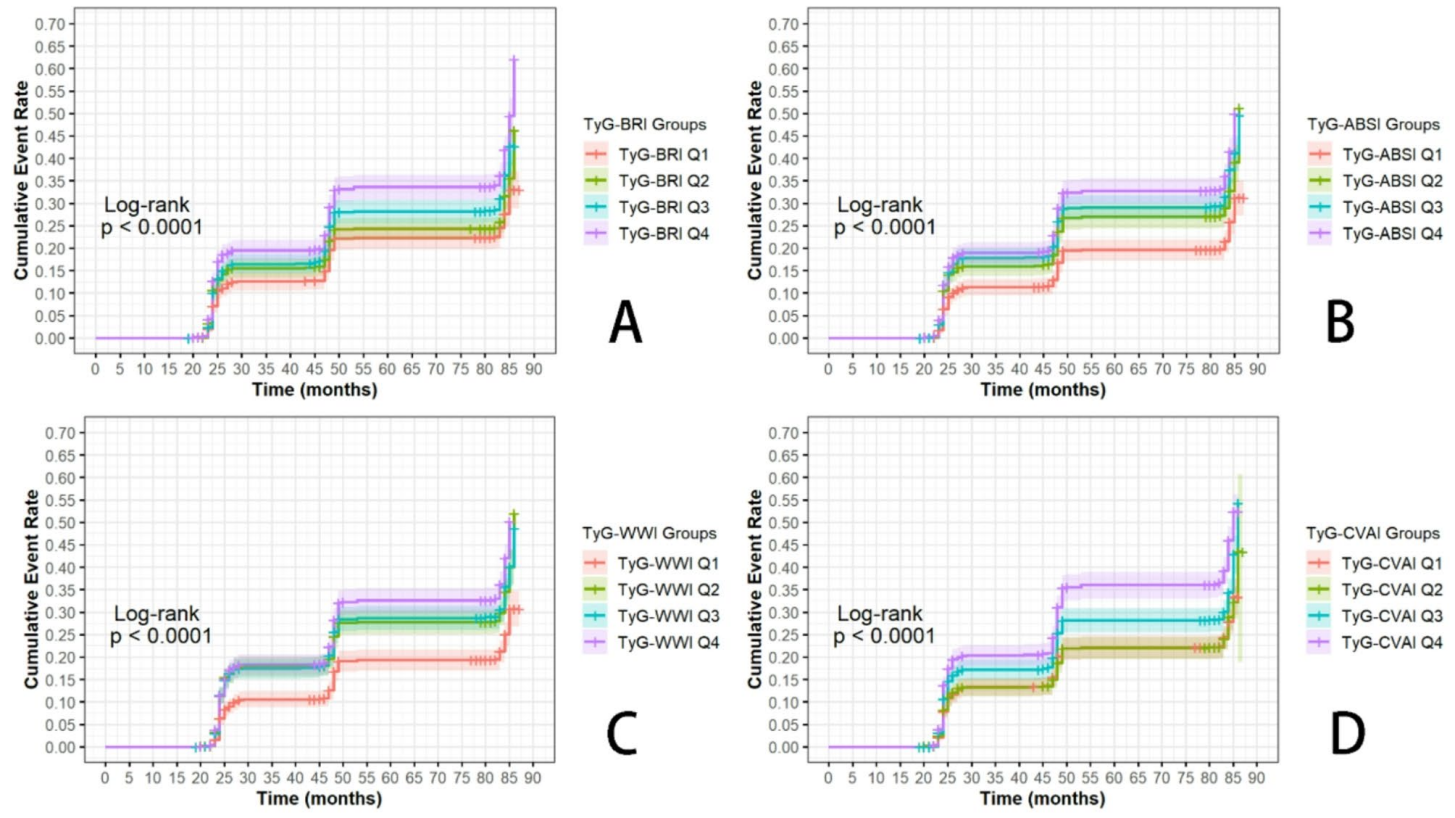
Kaplan–Meier curves for new-onset hypertension across quartiles of TyG-related indices: (**A**) TyG-BRI, (**B**) TyG-ABSI, (**C**) TyG-WWI, and (**D**) TyG-CVAI

Figure 2 presents Kaplan–Meier curves for TyG-related indices stratified by quartiles. For every index, higher quartiles were associated with a stepwise increase in hypertension incidence. The participants in the highest quartile (Q4) presented the greatest cumulative risk over time, whereas those in the lowest quartile (Q1) presented the lowest risk. Log-rank tests indicated significant differences across quartiles for all four indices ( *p* value< 0.001).

To assess the relationships between TyG-related indices and the risk of incident hypertension, we developed three Cox proportional hazards regression models (Table 2). According to the test result, the fully adjusted Cox models constructed using the four TyG-related indices all demonstrated favorable performance. All models passed the proportional hazards assumption test (Cox-ZPH test global *p* value > 0.05) and the goodness-of-fit test (Grønnesby-Borgan test *p* value > 0.05), while maintaining low prediction error (Brier scores all approximately 0.196). These results indicate that the models meet the prerequisites for statistical application, and the predictive performance of the four indices is very similar, all possessing reliable accuracy and consistency(Additional file 2 Table S1-S2, Figure S1-S3).

**Table 2:** Cox regression analysis of TyG-related indices and the risk of new-onset hypertension

	Model I	P-value	Model II	P-value	Model III	P-value
	HR (95% CI)		HR (95% CI)		HR (95% CI)	
TyG-BRI (per 1 SD)	1.24 (1.18–1.31)	< 0.001*	1.22 (1.15–1.30)	< 0.001*	1.21 (1.14–1.28)	< 0.001*
TyG-BRI Quartile						
Q1	Ref		Ref		Ref	
Q2	1.14 (0.98–1.34)	0.092	1.15 (0.99–1.35)	0.073	1.15 (0.98–1.35)	0.079
Q3	1.36 (1.17–1.59)	< 0.001*	1.37 (1.18–1.61)	< 0.001*	1.36 (1.16–1.60)	0.001*
Q4	1.66 (1.44–1.93)	< 0.001*	1.62 (1.38–1.91)	< 0.001*	1.58 (1.34–1.87)	< 0.001*
TyG-ABSI (per 1 SD)	1.22 (1.15–1.29)	< 0.001*	1.11 (1.05–1.18)	< 0.001*	1.09 (1.03–1.16)	0.005*
TyG-ABSI Quartile						
Q1	Ref		Ref		Ref	
Q2	1.38 (1.18–1.61)	< 0.001*	1.31 (1.12–1.53)	< 0.001*	1.29 (1.11–1.52)	0.001*
Q3	1.55 (1.33–1.81)	< 0.001*	1.40 (1.20–1.64)	< 0.001*	1.37 (1.17–1.61)	< 0.001*
Q4	1.82 (1.57–2.12)	< 0.001*	1.44 (1.23–1.69)	< 0.001*	1.37 (1.16–1.62)	< 0.001*
TyG-WWI (per 1 SD)	1.23 (1.17–1.31)	< 0.001*	1.14 (1.07–1.21)	< 0.001*	1.12 (1.05–1.19)	< 0.001*
TyG-WWI Quartile						
Q1	Ref		Ref		Ref	
Q2	1.49 (1.27–1.74)	< 0.001*	1.45 (1.24–1.69)	< 0.001*	1.44 (1.23–1.69)	< 0.001*
Q3	1.53 (1.31–1.79)	< 0.001*	1.43 (1.22–1.68)	< 0.001*	1.41 (1.20–1.66)	< 0.001*
Q4	1.88 (1.61–2.18)	< 0.001*	1.58 (1.35–1.88)	< 0.001*	1.52 (1.28–1.81)	< 0.001*
TyG-CVAI (per 1 SD)	1.29 (1.23–1.35)	< 0.001*	1.21 (1.15–1.28)	< 0.001*	1.21 (1.18–1.28)	< 0.001*
TyG-CVAI Quartile						
Q1	Ref		Ref		Ref	
Q2	1.01 (0.86–1.18)	0.930	1.00 (0.85–1.17)	0.953	1.00 (0.85–1.17)	0.979
Q3	1.32 (1.13–1.53)	< 0.001*	1.25 (1.07–1.46)	0.005*	1.25 (1.06–1.46)	0.006*
Q4	1.82 (1.57–2.10)	< 0.001*	1.58 (1.35–1.85)	< 0.001*	1.56 (1.32–1.85)	< 0.001*

Model 1, unadjusted; Model 2, adjusted for age, gender, residence, BMI, UA, TC, eGFR, HbA1c, education level, marital status, smoking status, and drinking status; Model 3, adjusted for variables included in Model 2 and heart disease, diabetes, dyslipidemia, stroke, lung disease, asthma and kidney disease

BMI: body mass index, eGFR: estimated glomerular filtration rate, UA: uric acid, HbA1c: glycated haemoglobin, TC: total cholesterol

In the unadjusted model (Model 1), each 1-SD increase in the TyG-BRI was associated with a 24% greater risk of hypertension (HR = 1.24, 95% CI: 1.18–1.31). This association remained significant after adjusting for age, gender, residence, BMI, TC, eGFR, HbA1c, UA, education level, marital status, smoking status, and drinking status in Model 2 (HR = 1.22, 95% CI: 1.15–1.30), and was slightly attenuated but still statistically significant in the fully adjusted model (Model 3), which accounted for heart disease, diabetes, dyslipidemia, stroke, lung disease, asthma and kidney disease (HR = 1.21, 95% CI: 1.14–1.28). When the TyG-BRI was examined as a categorical variable, participants in Q2, Q3, and Q4 had higher risks of hypertension than those in Q1 did, with adjusted hazard ratios of 1.15 (0.98–1.35), 1.36 (1.16–1.60), and 1.58 (1.34–1.87), respectively. Similarly, for the TyG-ABSI, each 1-SD increase was associated with a 22% greater risk of hypertension in Model 1 (HR = 1.22, 95% CI: 1.15–1.29), and the association remained significant in Model 3 after full adjustment (HR = 1.09, 95% CI: 1.03–1.16). In the quartile-based analysis, compared with Q1, Q2, Q3, and Q4 were significantly associated with an increased risk of hypertension, with HRs of 1.29 (95% CI: 1.11–1.52), 1.37 (95% CI: 1.17–1.61), and 1.37 (95% CI: 1.16–1.62), respectively. Notably, participants in Q4 presented a slightly lower risk than those in Q3 did. For the TyG-WWI, each 1-SD increase was associated with a 23% higher risk of hypertension in the unadjusted model (HR = 1.23, 95% CI: 1.17–1.31) and an 12% increase in the fully adjusted model (HR = 1.12, 95% CI: 1.05–1.19). In the quartile analysis, the adjusted HRs for Q2, Q3, and Q4 were 1.44 (95% CI: 1.23–1.69), 1.41 (95% CI: 1.20–1.66), and 1.52 (95% CI: 1.28–1.81), respectively. Similar to the trend observed for the TyG-ABSI, participants in Q3 presented a slightly lower risk than did those in Q2. For the TyG-CVAI, each 1-SD increase was associated with a 29% greater risk of hypertension in Model 1 (HR = 1.29, 95% CI: 1.23–1.35), which attenuated to 21% in Model 3 after full adjustment (HR = 1.21, 95% CI: 1.18–1.28). According to the categorical analysis, participants in Q3 and Q4 presented significantly greater risks than did those in Q1, with HRs of 1.25 (95% CI: 1.06–1.46) and 1.56 (95% CI: 1.32–1.85), respectively. Although participants in Q2 also exhibited a certain increase in cumulative risk, this increase was not statistically significant( *p* value= 0.979).

### Nonlinear associations between TyG-related indices and hypertension risk

**Fig. 3:**
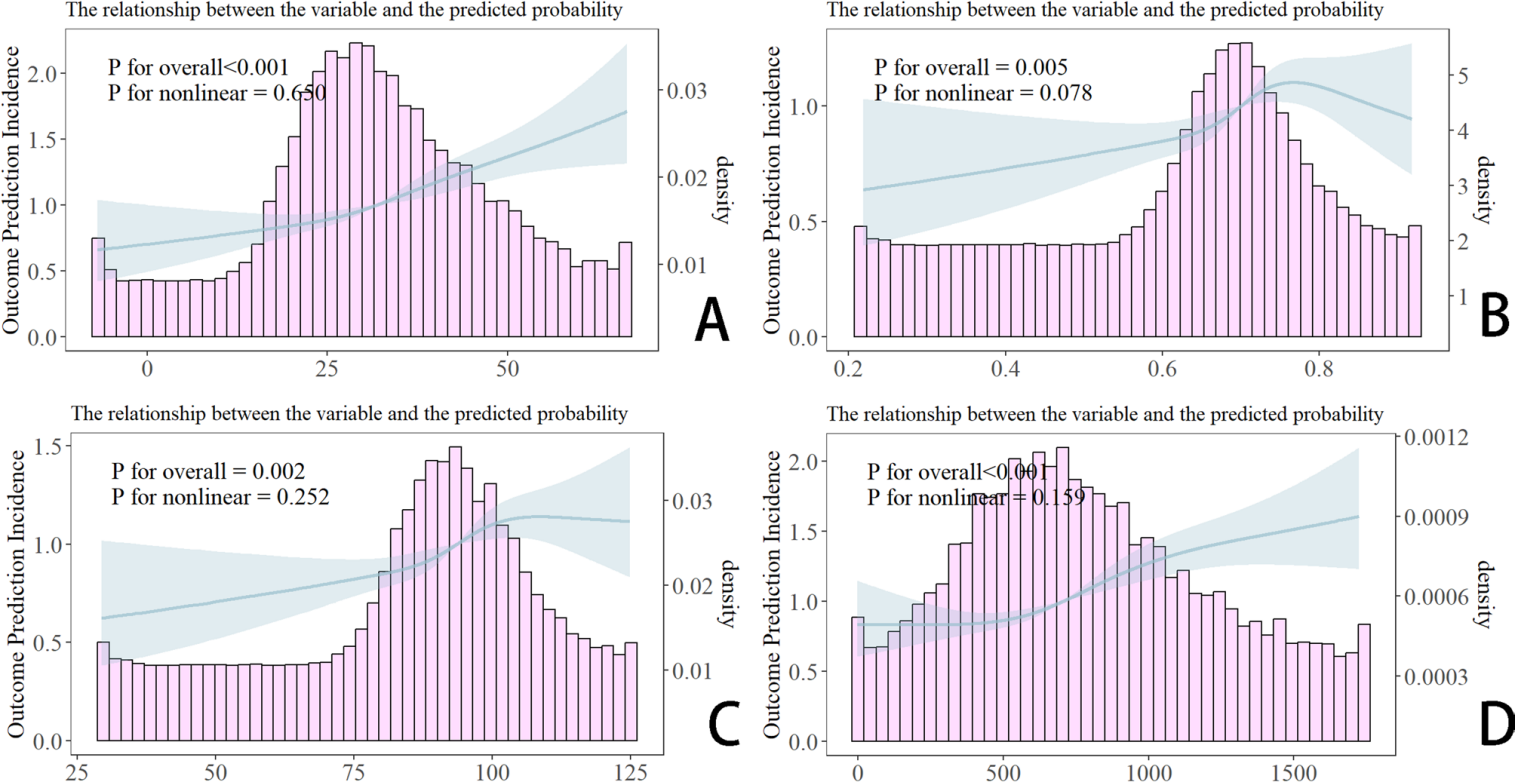
Restricted cubic spline curves illustrating the associations between TyG-related indices and new-onset hypertension risk. (**A**) TyG-BRI, (**B**) TyG-ABSI, (**C**) TyG-WWI, and (**D**) TyG-CVAI. The solid lines represent adjusted hazard ratios derived from multivariate Cox models, and the shaded areas denote 95% confidence intervals. Adjusted for age, gender, residence, BMI, eGFR, HbA1c, TC, UA, heart disease, diabetes, dyslipidemia, stroke, kidney disease, lung disease, asthma, education level, marital status, smoking status, drinking status

RCS analyses were conducted to evaluate potential nonlinear relationships between TyG-related indices and the risk of new-onset hypertension. As shown in Fig. 3, no evidence of nonlinearity was detected for the TyG-BRI, TyG-ABSI, TyG-WWI and TyG-CVAI (*p* value for nonlinearity > 0.05). The RCS curve indicated a potential change in the slope of the association between TyG-ABSI and incident hypertension, although the overall test for nonlinearity (Wald/likelihood-ratio test) was not statistically significant ( *p* value ≈ 0.08).

### Comparative predictive performance of TyG and related indices

**Fig. 4:**
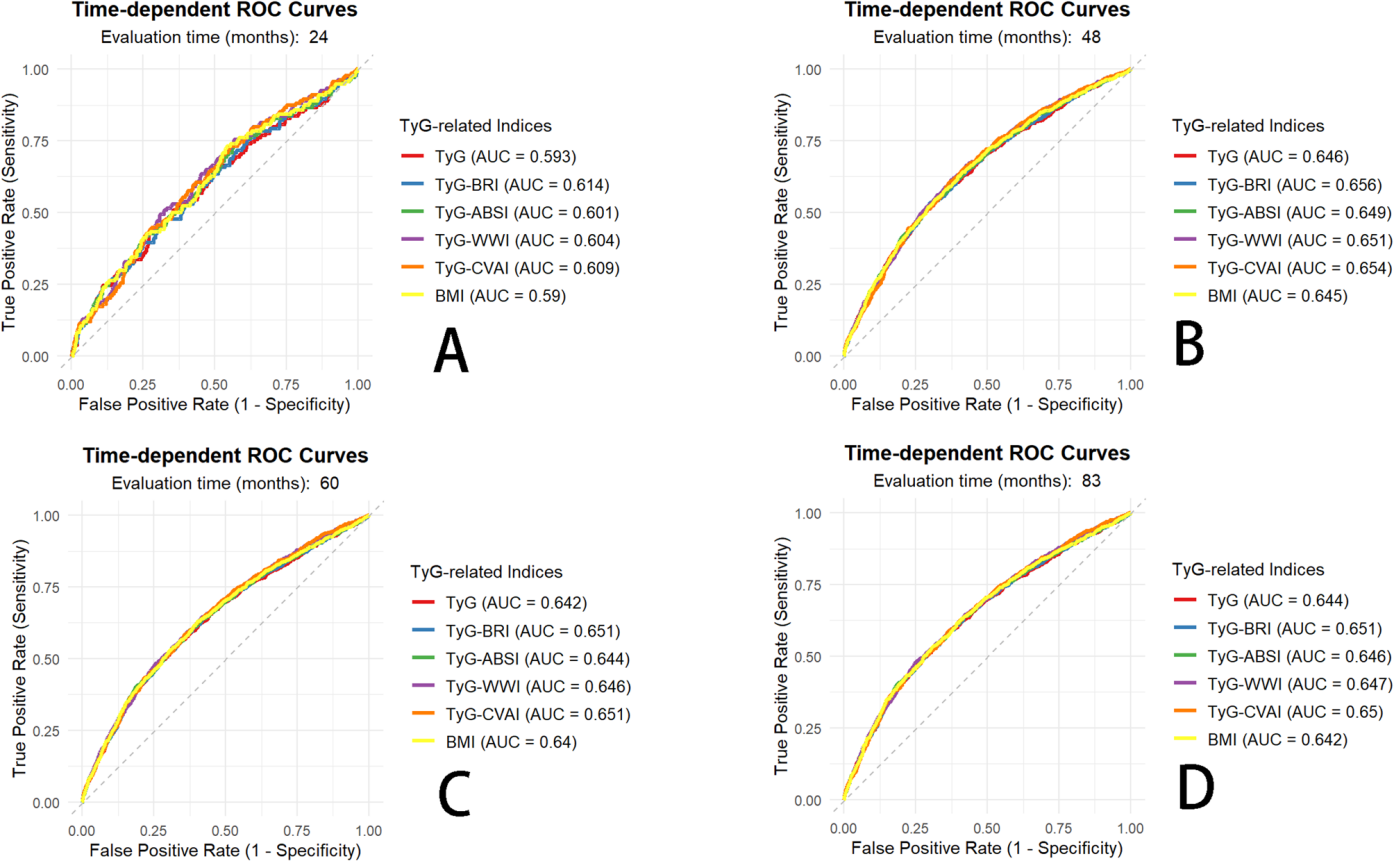
Time-dependent receiver operating characteristic (td-ROC) curves for the four indices at 24, 48, 60 and 83(median follow-up) months. BMI: base model including BMI (**A**) 24 months, (**B**) 48 months, (**C**) 60 months, and (**D**) 83 months

We applied Model 3 (the fully adjusted model) to construct time-dependent ROC curves. Time-dependent ROC curves were generated for six models—all based on the BMI-included base model, with the TyG index or each TyG-related index added separately. Based on the follow-up duration of the database and clinical relevance, we selected 24, 48, and 60 months, as well as the median follow-up time (83 months). (Fig. 4). As illustrated in Fig. 4, the areas under the ROC curves (AUCs) for the four TyG-related indices in predicting incident hypertension ranged from 0.60 to 0.66. The time-dependent AUCs of the TyG-related indices increased over time and then reached a stable plateau(Additional File 3 Figure S1). At all four time points, TyG-BRI consistently exhibited the highest AUCs, and the AUCs of all four composite indices were greater than those of TyG and base model including BMI.

The results of the DeLong test indicate that: during short-term follow-up (24 months), TyG-ABSI and TyG-WWI demonstrated statistically significant predictive performance in multiple comparisons ( *p* value <0.05). TyG-BRI showed significant or marginally significant differences with TyG index and base model at 24,48 and 60 months ( *p* value≈ 0.02–0.07), despite having the highest AUC, while most other comparisons were non-significant. At median follow-up (83 months), differences among all indices were non-significant, indicating convergence of predictive performance over time. The vast majority of comparisons among TyG-related indices were not statistically significant during follow-up (Additional file 3 Table S1).

### Net reclassification improvement (NRI) and integrated discrimination improvement (IDI) analysis

Based on the NRI and IDI results (Additional file 3 Table S2-S5), TyG-related indices generally demonstrated better predictive performance compared to both the baseline TyG index and base model including BMI across most follow-up periods. Specifically, TyG-BRI and TyG-CVAI showed relatively more stable improvements, with higher NRI and small but consistently positive IDI values (e.g., NRI around 0.11–0.17 and IDI ranging from 0.0001 to 0.0008). These two indices slightly enhanced the reclassification of non-events and overall discriminative ability, making them the relatively reliable predictors over time.

Based on the NRI results, while TyG-ABSI and TyG-WWI also provided significant improvements, their contributions were more modest and varied: TyG-ABSI improved event classification but performed poorly in non-event reclassification, whereas TyG-WWI showed minor but consistent gains. Overall, TyG-BRI and TyG-CVAI are recommended as replacements for TyG or base model in long-term risk prediction, offering enhanced accuracy and clinical utility.

### Subgroup analyses

**Fig. 5:**
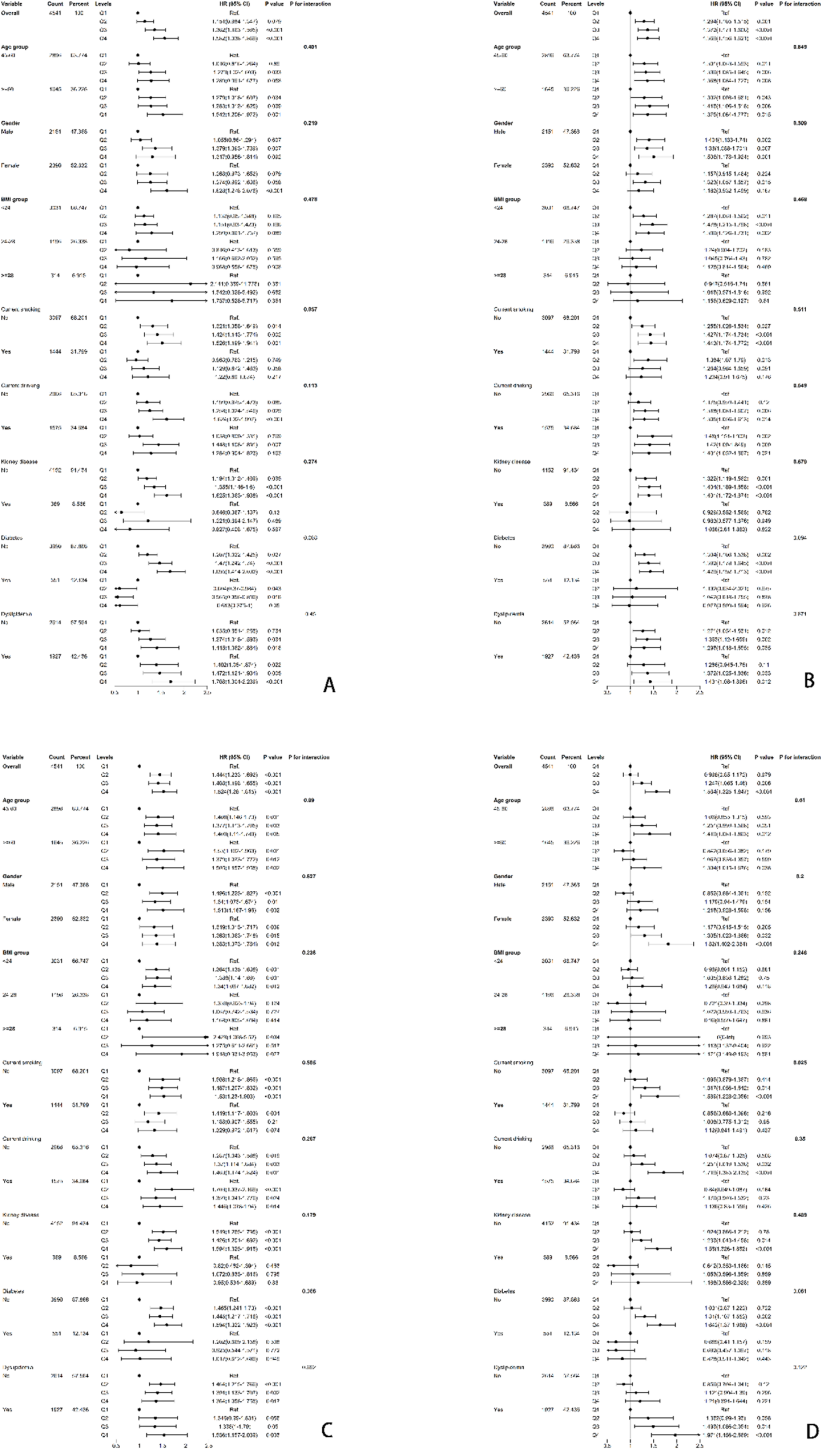
Subgroup analyses of the associations between TyG-related indices and new-onset hypertension. (**A**) TyG-BRI, (**B**) TyG-ABSI, (**C**) TyG-WWI, and (**D**) TyG-CVAI. Models were adjusted based on the basis of Model 3., with the stratification variable itself excluded from adjustment

To further investigate the relationships between the TyG-related indices and hypertension incidence, a series of subgroup analyses were conducted. In light of the eight subgroup analyses performed, a Bonferroni-corrected significance threshold of α = 0.00625 was applied. As shown in Fig. 5, none of the subgroups including age(< 60,≥60), gender, smoking status, drinking status, the prevalence of kidney disease, dyslipidemia, the prevalence of diabetes, and BMI(< 24,24–28,≥28), affected the relationships between the four TyG-related indices and hypertension incidence (all *p* values for interactions > 0.00625). After adjustment, none of the p-values reached statistical significance. Nonetheless, a potential signal was observed in the diabetic subgroup, suggesting that the application of TyG-related indices in individuals with diabetes should be interpreted with caution.

### Sensitivity analysis

Consistent outcomes were observed when various methods were employed to validate the robustness of the primary results in our study.

First, multiple imputation was performed for participants with missing covariates, and the analyses were conducted using the same Cox model as in the primary analysis. The findings remained largely robust (Additional file 4 Table S1).

Second, the association between TyG-related indices and new-onset hypertension was influenced by outliers, as evidenced by robust categorical analyses but unstable continuous variable models. (Additional file 4: Table S2).

Third, after individuals who had already developed hypertension at Wave 2 (2013) were excluded, the positive associations between TyG-related indices and the incidence of hypertension remained consistent. In multi-adjusted models, all TyG-combined indices were significantly associated with incident hypertension in a dose-response manner. The TyG-WWI emerged as the most stable indicator, with significantly elevated risk from Q2 onwards, while the TyG-CVAI best identified the highest-risk group (Q4). The TyG-BRI and TyG-ABSI showed less stability in lower quartiles. Overall, the TyG-WWI demonstrates superiority for reliable risk stratification. (Additional file 4: Table S3).

Finally, E-values were computed to quantify the minimum strength of association that an unmeasured confounder would need to have with both the exposure and the outcome to explain away the observed associations. (Additional file 4: Table S4)

## Discussion

This study, based on nationally representative data from the CHARLS cohort, is the first to systematically investigate the associations between four TyG-related indices (TyG-BRI, TyG-ABSI, TyG-WWI, and TyG-CVAI) and the risk of developing hypertension. The findings revealed that elevated levels of these indices were significantly associated with an increased risk of incident hypertension, even after adjusting for demographic characteristics, clinical variables, and metabolic covariates. Among them, TyG-CVAI and TyG-BRI demonstrated the strongest predictive performance. All indices showed a positive linear relationship with hypertension risk. A borderline non-linear relationship was observed between the TyG-ABSI index and incident hypertension risk (*p* value= 0.07). Subgroup analyses further confirmed these associations, particularly among participants without hypertension at baseline, where the associations were more pronounced. Collectively, these results highlight that TyG indices combined with obesity-related measures may serve as convenient and reliable biomarkers for identifying individuals at high risk of hypertension.

Obesity—particularly the accumulation of visceral fat—has been recognized as a major pathogenic factor for hypertension [22–24]. Substantial evidence indicates that insulin resistance and obesity exert complex, synergistic effects on the pathogenesis of hypertension through multiple pathways. Insulin resistance induces compensatory hyperinsulinemia, which activates several renal sodium transporters, including NHE3, NKCC2, and ENaC. This activation increases renal sodium reabsorption, leading to sodium and water retention, expanded blood volume, and elevated blood pressure [25–31].

In the insulin-resistant state, the PI3K–Akt–eNOS signaling pathway is impaired, resulting in decreased nitric oxide (NO) production, while the MAPK pathway is upregulated, promoting the secretion of endothelin-1 (ET-1). The disruption of the NO/ET-1 balance enhances vasoconstriction, increases vascular resistance, and stimulates vascular smooth muscle cell proliferation [32–35].

Moreover, during obesity, adipose tissue–derived hormones such as leptin activate the hypothalamic melanocortin system. Together with other mechanisms—including chemoreflex activation and impaired baroreflex sensitivity—this leads to sustained sympathetic overactivity. Consequently, heart rate and myocardial contractility increase, renal vasoconstriction intensifies, and renin release is stimulated, collectively contributing to elevated blood pressure [36–38].

Although body mass index (BMI) and waist circumference are widely used in clinical and epidemiological studies, their ability to accurately assess abdominal fat distribution is limited. In response, several novel obesity-related indices—such as the Body Roundness Index (BRI), A Body Shape Index (ABSI), Weight-Adjusted Waist Index (WWI), and Chinese Visceral Adiposity Index (CVAI)—have been developed. These indices provide a more precise evaluation of visceral fat accumulation and have demonstrated superior performance in predicting the risk of cardiovascular and cerebrovascular diseases.

However, research exploring their associations with hypertension remains limited. A longitudinal research from Mexico reported that individuals in the high TyG group had a 56% increased risk of developing hypertension compared with those in the low TyG group (HR = 1.56, 95% CI: 1.18–2.08) [39]. Yang Chen et al. revealed that the relationship between the triglyceride–glucose (TyG) index and hypertension risk varies significantly depending on waist circumference, but their analysis did not account for the influence of height on abdominal obesity [40]. In the present study, we incorporated composite indices such as TyG-BRI and TyG-CVAI to enhance the predictive ability for identifying individuals at risk of developing hypertension.

The CVAI, developed by Xia et al. in 2016, integrates waist circumference, BMI, age, and sex to estimate visceral fat area specifically for the Chinese population [41]. The BRI, proposed by Thomas et al. in 2013, is based on an elliptical body model that uses body eccentricity to estimate visceral and total body fat percentages [42].

Given the well-established interaction between obesity and hypertension under insulin-resistant conditions, these findings may help explain why the composite indices incorporating both insulin resistance and visceral adiposity (TyG-CVAI and TyG-BRI) demonstrated superior predictive capability.

Although all four indices were identified as independent risk factors for incident hypertension, the predictive performance of TyG-ABSI was relatively weaker. This finding is consistent with the study by Calderón-García et al. [43], and may be attributed to the fact that ABSI—originally developed in Western populations as a novel index for predicting mortality [44]—has not yet been thoroughly validated for predicting hypertension incidence in the CHARLS cohort.

TyG-WWI, which adjusts waist circumference solely by body weight, overlooks the influence of height and other factors on central obesity, resulting in slightly weaker predictive performance. In contrast, TyG-CVAI incorporates sex as a parameter, and its superior predictive ability may reflect the interplay between insulin resistance and the sex hormone–visceral fat axis.

The potential mechanism aligns with the “low testosterone–obesity–adipocytokine hypothesis” proposed by Jones [45]. In this model, adipose tissue overexpresses aromatase, which converts testosterone into estradiol, leading to a state of low testosterone. Reduced testosterone levels increase lipoprotein lipase activity and stimulate pluripotent stem cells to differentiate into adipocytes, thereby promoting triglyceride storage and exacerbating central obesity. The resulting accumulation of adipose mass is closely linked to insulin resistance, further aggravating metabolic dysfunction.

In addition, estradiol and adipocytokines (such as IL-6 and leptin) can suppress the hypothalamic–pituitary axis, further reducing testosterone secretion and creating a vicious cycle.

In our study, we conducted time-dependent ROC analyses to dynamically evaluate the predictive value of TyG-based obesity indices for incident hypertension. To our knowledge, this is the first study to validate their predictive performance within a temporal framework. The trend plots clearly demonstrated that all indices exhibited their highest predictive ability during the early follow-up period (the first 2–3 years), after which the AUC(t) values gradually stabilized or slightly declined over time.

Furthermore, we comprehensively assessed the predictive performance of the TyG index and its derived composite indices (TyG-BRI, TyG-ABSI, TyG-WWI, and TyG-CVAI) using DeLong’s test and the Net Reclassification Improvement (NRI) metric. Our findings revealed that the composite indices—particularly TyG-BRI and TyG-CVAI—showed better discrimination and reclassification ability compared with the conventional TyG index and base model during the mid-term follow-up (24–60 months). This advantage appeared to be time-dependent, as the differences diminished during long-term follow-up (83 months).

Moreover, the mechanisms underlying these improvements varied across indices. For instance, the superiority of TyG-CVAI and TyG-BRI primarily stemmed from their improvements in identifying non-events (participants who did not develop hypertension), whereas TyG-ABSI demonstrated better event identification but with reduced non-event identification.

Our findings hold important clinical and public health implications. In the primary prevention of hypertension, early identification of high-risk individuals is crucial for timely intervention. As low-cost and noninvasive calculated indices, TyG-CVAI and TyG-BRI can be effectively applied in large-scale population screening, particularly in regions with limited medical resources. These indices enable clinicians to more accurately identify individuals who would most benefit from intensified lifestyle interventions during the critical window before the onset of hypertension. Such targeted prevention strategies could greatly enhance the efficiency and cost-effectiveness of public health efforts.

### Strengths and limitations

This study possesses several notable strengths. First, it was based on the nationally representative, large-scale prospective CHARLS cohort, which provides extensive population coverage and enhances the generalizability and external validity of the findings. Second, this research is the first to systematically and concurrently compare four TyG-related composite indices in relation to the risk of incident hypertension, thereby addressing a gap in the existing literature and underscoring its originality. Methodologically, the study employed a rigorous analytical framework—comprehensively adjusting for potential confounders through multivariable modeling, validating the robustness of results via subgroup analyses, and innovatively incorporating time-dependent ROC curves and the Net Reclassification Improvement (NRI) index. These approaches allowed for an in-depth dynamic evaluation of predictive performance across time, particularly identifying the optimal predictive window (24–60 months) for TyG-BRI and TyG-CVAI. Collectively, these methodological strengths highlight both the scientific depth and the practical value of this study in informing precision prevention strategies. However, several limitations should also be acknowledged.

First, the TyG and obesity-related indices were derived from baseline measurements only, preventing assessment of their longitudinal changes and potential dynamic associations with hypertension risk. Although hypertension outcomes were determined using multiple criteria, blood pressure data were only available for the first three survey waves, with the fourth relying primarily on self-reported information—an approach that may have introduced misclassification, despite the established reliability of CHARLS data. Second, as an observational study, residual confounding from unmeasured variables cannot be entirely excluded, even after extensive adjustment, and causal inferences cannot be established without further experimental or longitudinal validation. Additionally, the CHARLS cohort includes only middle-aged and older Chinese adults; thus, extrapolation to younger populations or other ethnic groups should be approached with caution. Owing to the lack of an independent validation cohort within the CHARLS framework, external validation was not feasible.

Finally, the predictive advantage of the composite indices demonstrated clear time dependence, with diminishing differences observed during long-term follow-up. This finding suggests that the clinical application of these indices should consider their optimal predictive window. Future studies could explore more sophisticated longitudinal modeling approaches or integrate emerging biomarkers to develop predictive tools with stable performance across the entire follow-up period.

Future studies should aim to validate these associations in cohorts with greater ethnic and age diversity, and explore how TyG-related indices evolve over time in relation to hypertension risk. Research based on different diagnostic criteria for hypertension is also warranted. In addition, mechanistic studies are needed to elucidate how insulin resistance and central obesity interact to promote the development of hypertension. Ultimately, incorporating TyG-related indices into strategies for hypertension prevention and management may be beneficial, particularly in middle-aged and older populations, where improving insulin resistance and central obesity could help reduce the risk of hypertension.

## Conclusion

TyG-BRI, TyG-ABSI, TyG-WWI, and TyG-CVAI were each independently associated with a higher risk of incident hypertension. These findings support the utility of TyG-related indices for risk stratification and prevention of hypertension among middle-aged and older Chinese adults.

## Supplementary Information

Below is the link to the electronic supplementary material.

Supplementary Material 1

Supplementary Material 2

Supplementary Material 3

Supplementary Material 4

## Data Availability

The dataset utilized in this study is publicly available from the official CHARLS website (https://charls.pku.edu.cn/). Upon registration and approval, users may access and download the data in accordance with the platform’s guidelines.
