# Supplementary material for: Associations between novel triglyceride-glucose-related indices and the incidence of hypertension among Chinese middle-aged and elderly adults: a nationwide prospective cohort study
Source: Cardiovasc Diabetol Endocrinol Rep. 2025 Dec 29;11:50. doi: 10.1186/s40842-025-00255-3 (PMC12746625; doi:10.1186/s40842-025-00255-3)
Supplement: Supplementary file 1 — Supplementary Material 1 [file 40842_2025_255_MOESM1_ESM.docx]

**Table S1 Baseline Characteristics of participants according to quartiles of TyG-BRI**

|  | **[ALL]** | **Q1** | **Q2** | **Q3** | **Q4** | **p.overall** |
| --- | --- | --- | --- | --- | --- | --- |
| Variables | ***N=4541*** | ***N=1123*** | ***N=1152*** | ***N=1137*** | ***N=1129*** |  |
| Age,years | 56.00 [50.00;63.00] | 56.00 [50.00;63.00] | 56.00 [51.00;63.00] | 56.00 [49.00;62.00] | 57.00 [51.00;63.00] | 0.400 |
| Gender,n% |  |  |  |  |  | <0.001 |
| Male | 2151 (47.37%) | 731 (65.09%) | 643 (55.82%) | 473 (41.60%) | 304 (26.93%) |  |
| Female | 2390 (52.63%) | 392 (34.91%) | 509 (44.18%) | 664 (58.40%) | 825 (73.07%) |  |
| Marry,n% |  |  |  |  |  | 0.257 |
| Yes | 3923 (86.39%) | 983 (87.53%) | 992 (86.11%) | 990 (87.07%) | 958 (84.85%) |  |
| Education,n% |  |  |  |  |  | 0.375 |
| Below middle school | 3071 (67.63%) | 752 (66.96%) | 758 (65.80%) | 774 (68.07%) | 787 (69.71%) |  |
| Middle school and high school | 1419 (31.25%) | 358 (31.88%) | 377 (32.73%) | 350 (30.78%) | 334 (29.58%) |  |
| College or higher | 51 (1.12%) | 13 (1.16%) | 17 (1.48%) | 13 (1.14%) | 8 (0.71%) |  |
| Residence,n% |  |  |  |  |  | <0.001 |
| Urban | 1530 (33.69%) | 310 (27.60%) | 355 (30.82%) | 419 (36.85%) | 446 (39.50%) |  |
| Rural | 3011 (66.31%) | 813 (72.40%) | 797 (69.18%) | 718 (63.15%) | 683 (60.50%) |  |
| BMI (kg/m2) | 22.53 [20.41;24.82] | 19.82 [18.58;21.17] | 21.66 [20.29;22.89] | 23.43 [21.93;24.69] | 26.15 [24.45;27.96] | 0.000 |
| Drinking,n% |  |  |  |  |  | <0.001 |
| Never | 2652 (58.40%) | 555 (49.42%) | 618 (53.65%) | 697 (61.30%) | 782 (69.26%) |  |
| Current | 1575 (34.68%) | 482 (42.92%) | 438 (38.02%) | 373 (32.81%) | 282 (24.98%) |  |
| Former | 314 (6.91%) | 86 (7.66%) | 96 (8.33%) | 67 (5.89%) | 65 (5.76%) |  |
| Smoking,n% |  |  |  |  |  | <0.001 |
| Never | 2741 (60.36%) | 508 (45.24%) | 625 (54.25%) | 763 (67.11%) | 845 (74.84%) |  |
| Now | 1444 (31.80%) | 533 (47.46%) | 428 (37.15%) | 283 (24.89%) | 200 (17.71%) |  |
| Former | 356 (7.84%) | 82 (7.30%) | 99 (8.59%) | 91 (8.00%) | 84 (7.44%) |  |
| Diabetes,n%: |  |  |  |  |  | <0.001 |
| No | 3990 (87.87%) | 1049 (93.41%) | 1055 (91.58%) | 993 (87.34%) | 893 (79.10%) |  |
| Yes | 551 (12.13%) | 74 (6.59%) | 97 (8.42%) | 144 (12.66%) | 236 (20.90%) |  |
| Dyslipidemia,n%: |  |  |  |  |  | <0.001 |
| No | 2614 (57.56%) | 849 (75.60%) | 765 (66.41%) | 584 (51.36%) | 416 (36.85%) |  |
| Yes | 1927 (42.44%) | 274 (24.40%) | 387 (33.59%) | 553 (48.64%) | 713 (63.15%) |  |
| Heart disease,n%: |  |  |  |  |  | 0.004 |
| No | 4117 (90.66%) | 1036 (92.25%) | 1058 (91.84%) | 1027 (90.33%) | 996 (88.22%) |  |
| Yes | 424 (9.34%) | 87 (7.75%) | 94 (8.16%) | 110 (9.67%) | 133 (11.78%) |  |
| Kidney disease,n% |  |  |  |  |  | 0.645 |
| No | 4152 (91.43%) | 1028 (91.54%) | 1043 (90.54%) | 1044 (91.82%) | 1037 (91.85%) |  |
| Yes | 389 (8.57%) | 95 (8.46%) | 109 (9.46%) | 93 (8.18%) | 92 (8.15%) |  |
| Stroke,n% |  |  |  |  |  | 0.076 |
| No | 4481 (98.68%) | 1108 (98.66%) | 1142 (99.13%) | 1125 (98.94%) | 1106 (97.96%) |  |
| Yes | 60 (1.32%) | 15 (1.34%) | 10 (0.87%) | 12 (1.06%) | 23 (2.04%) |  |
| Lung disease,n% |  |  |  |  |  | 0.053 |
| No | 4032 (88.79%) | 975 (86.82%) | 1021 (88.63%) | 1028 (90.41%) | 1008 (89.28%) |  |
| Yes | 509 (11.21%) | 148 (13.18%) | 131 (11.37%) | 109 (9.59%) | 121 (10.72%) |  |
| Asthma,n% |  |  |  |  |  | 0.311 |
| No | 4343 (95.64%) | 1067 (95.01%) | 1104 (95.83%) | 1097 (96.48%) | 1075 (95.22%) |  |
| Yes | 198 (4.36%) | 56 (4.99%) | 48 (4.17%) | 40 (3.52%) | 54 (4.78%) |  |
| SBP(mmHg) | 118.33 [109.67;127.33] | 115.67 [107.33;125.50] | 117.33 [109.00;126.00] | 118.67 [110.33;127.33] | 121.67 [113.00;129.33] | <0.001 |
| DBP(mmHg) | 70.33 [64.33;76.67] | 69.00 [62.67;75.17] | 69.33 [63.33;76.00] | 71.00 [64.67;77.00] | 72.67 [66.33;78.67] | <0.001 |
| eGFR | 96.56 [86.90;104.03] | 96.98 [88.05;104.58] | 96.39 [87.28;103.32] | 97.10 [86.67;104.46] | 96.42 [85.51;103.31] | 0.058 |
| FBG (mg/dl) | 100.80 [93.42;109.98] | 97.20 [90.54;105.93] | 99.63 [92.52;107.82] | 101.52 [94.50;109.80] | 105.12 [96.48;117.72] | <0.001 |
| TG (mg/dl) | 96.46 [70.80;139.83] | 75.22 [57.53;101.33] | 86.73 [66.38;118.81] | 104.43 [77.88;143.37] | 134.52 [97.35;197.35] | <0.001 |
| Scr (mg/dl) | 0.75 [0.64;0.87] | 0.78 [0.67;0.89] | 0.77 [0.66;0.88] | 0.73 [0.63;0.86] | 0.71 [0.62;0.84] | <0.001 |
| UA (mg/dl) | 4.17 [3.49;4.95] | 4.17 [3.47;4.88] | 4.15 [3.51;4.97] | 4.15 [3.45;4.96] | 4.20 [3.53;4.99] | 0.481 |
| BUN (mg/dl) | 15.07 [12.49;18.07] | 15.63 [12.83;18.71] | 15.08 [12.55;18.23] | 15.10 [12.38;17.95] | 14.71 [12.32;17.37] | <0.001 |
| LDL-C (mg/dl) | 114.05 [93.17;135.70] | 107.09 [88.92;127.58] | 111.34 [91.62;132.22] | 117.53 [96.26;139.18] | 119.46 [96.65;141.50] | <0.001 |
| HDL-C (mg/dl) | 51.03 [41.75;61.47] | 56.83 [46.78;67.08] | 53.74 [44.85;63.79] | 49.48 [40.98;58.76] | 44.46 [36.34;53.35] | <0.001 |
| HbA1c (%) | 5.10 [4.90;5.40] | 5.10 [4.80;5.30] | 5.10 [4.80;5.40] | 5.10 [4.90;5.40] | 5.20 [5.00;5.60] | <0.001 |
| TC (mg/dl) | 188.66 [165.85;213.40] | 179.38 [158.89;205.09] | 184.99 [164.21;209.15] | 192.14 [167.78;214.95] | 196.78 [173.97;222.29] | <0.001 |
| TyG | 8.50 [8.15;8.91] | 8.21 [7.94;8.54] | 8.38 [8.07;8.72] | 8.59 [8.28;8.94] | 8.90 [8.50;9.33] | <0.001 |
| BRI | 3.74 [3.01;4.67] | 2.57 [2.23;2.81] | 3.38 [3.18;3.60] | 4.15 [3.90;4.43] | 5.38 [4.96;5.95] | 0.000 |
| TyG-BRI | 31.87 [25.11;40.61] | 21.14 [18.29;23.17] | 28.50 [26.73;30.02] | 35.83 [33.72;38.01] | 47.74 [43.97;53.42] | 0.000 |
| ABSI | 0.08 [0.08;0.09] | 0.08 [0.08;0.08] | 0.08 [0.08;0.08] | 0.08 [0.08;0.09] | 0.08 [0.08;0.09] | <0.001 |
| TyG-ABSI | 0.70 [0.66;0.75] | 0.65 [0.61;0.68] | 0.69 [0.65;0.72] | 0.72 [0.68;0.76] | 0.76 [0.72;0.81] | 0.000 |
| WWI | 10.98 [10.45;11.54] | 10.20 [9.85;10.51] | 10.77 [10.46;11.14] | 11.17 [10.87;11.57] | 11.75 [11.36;12.20] | 0.000 |
| TyG-WWI | 93.62 [86.88;100.95] | 83.44 [79.31;87.60] | 90.64 [86.89;94.51] | 96.53 [92.34;101.39] | 104.48 [99.92;111.33] | 0.000 |
| CVAI | 83.28 [60.09;110.18] | 46.65 [32.25;59.53] | 72.66 [61.65;84.06] | 95.14 [82.13;109.99] | 125.53 [109.28;145.51] | 0.000 |
| TyG-CVAI | 706.41 [497.80;962.03] | 383.89 [259.20;492.19] | 612.76 [510.50;712.45] | 825.34 [702.45;961.39] | 1117.28 [954.24;1333.12] | 0.000 |

**Table S2 Baseline Characteristics of participants according to quartiles of TyG-ABSI**

|  | **[ALL]** | **Q1** | **Q2** | **Q3** | **Q4** | **p.overall** |
| --- | --- | --- | --- | --- | --- | --- |
| Variables | ***N=4541*** | ***N=1139*** | ***N=1133*** | ***N=1141*** | ***N=1128*** |  |
| Age,years | 56.00 [50.00;63.00] | 54.00 [48.00;60.00] | 56.00 [50.00;62.00] | 57.00 [51.00;64.00] | 59.00 [53.00;65.00] | <0.001 |
| Gender,n% |  |  |  |  |  | <0.001 |
| Male | 2151 (47.37%) | 597 (52.41%) | 592 (52.25%) | 526 (46.10%) | 436 (38.65%) |  |
| Female | 2390 (52.63%) | 542 (47.59%) | 541 (47.75%) | 615 (53.90%) | 692 (61.35%) |  |
| Marry,n% |  |  |  |  |  | 0.076 |
| Yes | 3923 (86.39%) | 992 (87.09%) | 996 (87.91%) | 984 (86.24%) | 951 (84.31%) |  |
| Education,n% |  |  |  |  |  | <0.001 |
| Below middle school | 3071 (67.63%) | 726 (63.74%) | 750 (66.20%) | 773 (67.75%) | 822 (72.87%) |  |
| Middle school and high school | 1419 (31.25%) | 396 (34.77%) | 375 (33.10%) | 352 (30.85%) | 296 (26.24%) |  |
| College or higher | 51 (1.12%) | 17 (1.49%) | 8 (0.71%) | 16 (1.40%) | 10 (0.89%) |  |
| Residence,n% |  |  |  |  |  | 0.004 |
| Urban | 1530 (33.69%) | 342 (30.03%) | 386 (34.07%) | 382 (33.48%) | 420 (37.23%) |  |
| Rural | 3011 (66.31%) | 797 (69.97%) | 747 (65.93%) | 759 (66.52%) | 708 (62.77%) |  |
| BMI (kg/m2) | 22.53 [20.41;24.82] | 21.99 [20.19;23.95] | 22.27 [20.28;24.44] | 22.85 [20.49;25.19] | 23.16 [20.72;25.68] | <0.001 |
| Drinking,n% |  |  |  |  |  | <0.001 |
| Never | 2652 (58.40%) | 614 (53.91%) | 641 (56.58%) | 703 (61.61%) | 694 (61.52%) |  |
| Current | 1575 (34.68%) | 452 (39.68%) | 410 (36.19%) | 347 (30.41%) | 366 (32.45%) |  |
| Former | 314 (6.91%) | 73 (6.41%) | 82 (7.24%) | 91 (7.98%) | 68 (6.03%) |  |
| Smoking,n% |  |  |  |  |  | <0.001 |
| Never | 2741 (60.36%) | 670 (58.82%) | 633 (55.87%) | 694 (60.82%) | 744 (65.96%) |  |
| Now | 1444 (31.80%) | 394 (34.59%) | 406 (35.83%) | 347 (30.41%) | 297 (26.33%) |  |
| Former | 356 (7.84%) | 75 (6.58%) | 94 (8.30%) | 100 (8.76%) | 87 (7.71%) |  |
| Diabetes,n%: |  |  |  |  |  | <0.001 |
| No | 3990 (87.87%) | 1086 (95.35%) | 1060 (93.56%) | 1017 (89.13%) | 827 (73.32%) |  |
| Yes | 551 (12.13%) | 53 (4.65%) | 73 (6.44%) | 124 (10.87%) | 301 (26.68%) |  |
| Dyslipidemia,n%: |  |  |  |  |  | <0.001 |
| No | 2614 (57.56%) | 914 (80.25%) | 767 (67.70%) | 601 (52.67%) | 332 (29.43%) |  |
| Yes | 1927 (42.44%) | 225 (19.75%) | 366 (32.30%) | 540 (47.33%) | 796 (70.57%) |  |
| Heart disease,n%: |  |  |  |  |  | <0.001 |
| No | 4117 (90.66%) | 1051 (92.27%) | 1040 (91.79%) | 1038 (90.97%) | 988 (87.59%) |  |
| Yes | 424 (9.34%) | 88 (7.73%) | 93 (8.21%) | 103 (9.03%) | 140 (12.41%) |  |
| Kidney disease,n% |  |  |  |  |  | 0.638 |
| No | 4152 (91.43%) | 1050 (92.19%) | 1034 (91.26%) | 1045 (91.59%) | 1023 (90.69%) |  |
| Yes | 389 (8.57%) | 89 (7.81%) | 99 (8.74%) | 96 (8.41%) | 105 (9.31%) |  |
| Stroke,n% |  |  |  |  |  | 0.083 |
| No | 4481 (98.68%) | 1128 (99.03%) | 1118 (98.68%) | 1130 (99.04%) | 1105 (97.96%) |  |
| Yes | 60 (1.32%) | 11 (0.97%) | 15 (1.32%) | 11 (0.96%) | 23 (2.04%) |  |
| Lung disease,n% |  |  |  |  |  | 0.227 |
| No | 4032 (88.79%) | 1027 (90.17%) | 1007 (88.88%) | 1012 (88.69%) | 986 (87.41%) |  |
| Yes | 509 (11.21%) | 112 (9.83%) | 126 (11.12%) | 129 (11.31%) | 142 (12.59%) |  |
| Asthma,n% |  |  |  |  |  | 0.250 |
| No | 4343 (95.64%) | 1095 (96.14%) | 1091 (96.29%) | 1088 (95.35%) | 1069 (94.77%) |  |
| Yes | 198 (4.36%) | 44 (3.86%) | 42 (3.71%) | 53 (4.65%) | 59 (5.23%) |  |
| SBP(mmHg) | 118.33 [109.67;127.33] | 116.33 [108.00;124.67] | 117.33 [109.33;126.00] | 119.00 [110.67;128.33] | 121.00 [111.67;129.00] | <0.001 |
| DBP(mmHg) | 70.33 [64.33;76.67] | 69.33 [63.33;76.00] | 70.33 [64.00;75.67] | 71.00 [65.00;77.33] | 71.33 [65.33;77.33] | <0.001 |
| eGFR | 96.56 [86.90;104.03] | 98.94 [90.01;105.97] | 96.81 [88.04;104.04] | 96.31 [85.50;103.31] | 94.44 [83.81;102.15] | <0.001 |
| FBG (mg/dl) | 100.80 [93.42;109.98] | 95.94 [89.37;103.50] | 98.82 [92.34;106.20] | 101.70 [94.50;109.80] | 108.72 [99.14;124.38] | <0.001 |
| TG (mg/dl) | 96.46 [70.80;139.83] | 64.61 [52.22;81.42] | 85.85 [69.92;106.20] | 108.86 [87.61;141.60] | 162.84 [115.93;230.99] | 0.000 |
| Scr (mg/dl) | 0.75 [0.64;0.87] | 0.75 [0.63;0.86] | 0.76 [0.66;0.87] | 0.73 [0.63;0.88] | 0.75 [0.64;0.86] | 0.311 |
| UA (mg/dl) | 4.17 [3.49;4.95] | 4.08 [3.40;4.80] | 4.15 [3.45;4.89] | 4.16 [3.54;4.97] | 4.31 [3.56;5.19] | <0.001 |
| BUN (mg/dl) | 15.07 [12.49;18.07] | 15.49 [12.61;18.48] | 15.07 [12.49;17.98] | 15.04 [12.52;17.95] | 14.82 [12.38;17.87] | 0.021 |
| LDL-C (mg/dl) | 114.05 [93.17;135.70] | 107.47 [89.69;127.58] | 114.05 [94.33;135.31] | 117.91 [97.04;138.79] | 117.14 [92.40;141.11] | <0.001 |
| HDL-C (mg/dl) | 51.03 [41.75;61.47] | 57.22 [49.10;67.46] | 52.58 [44.46;62.24] | 49.10 [40.59;59.15] | 44.07 [35.47;54.12] | <0.001 |
| HbA1c (%) | 5.10 [4.90;5.40] | 5.10 [4.80;5.30] | 5.10 [4.90;5.40] | 5.20 [4.90;5.40] | 5.20 [5.00;5.60] | <0.001 |
| TC (mg/dl) | 188.66 [165.85;213.40] | 177.84 [158.51;201.61] | 184.79 [163.92;208.38] | 190.59 [169.33;213.02] | 201.42 [177.06;230.51] | <0.001 |
| TyG | 8.50 [8.15;8.91] | 8.05 [7.81;8.27] | 8.38 [8.15;8.58] | 8.64 [8.42;8.90] | 9.12 [8.78;9.57] | 0.000 |
| BRI | 3.74 [3.01;4.67] | 2.96 [2.43;3.59] | 3.54 [2.93;4.24] | 3.99 [3.35;4.86] | 4.64 [3.87;5.46] | <0.001 |
| TyG-BRI | 31.87 [25.11;40.61] | 23.57 [19.42;28.73] | 29.69 [24.75;35.56] | 34.62 [28.96;42.05] | 42.46 [35.26;50.91] | 0.000 |
| ABSI | 0.08 [0.08;0.09] | 0.08 [0.07;0.08] | 0.08 [0.08;0.08] | 0.08 [0.08;0.09] | 0.09 [0.08;0.09] | 0.000 |
| TyG-ABSI | 0.70 [0.66;0.75] | 0.63 [0.60;0.64] | 0.68 [0.67;0.69] | 0.72 [0.71;0.73] | 0.79 [0.77;0.82] | 0.000 |
| WWI | 10.98 [10.45;11.54] | 10.28 [9.85;10.63] | 10.81 [10.42;11.19] | 11.20 [10.81;11.62] | 11.73 [11.23;12.25] | 0.000 |
| TyG-WWI | 93.62 [86.88;100.95] | 82.68 [78.97;85.49] | 90.33 [88.00;93.15] | 96.86 [94.10;99.95] | 106.60 [102.80;112.02] | 0.000 |
| CVAI | 83.28 [60.09;110.18] | 55.85 [36.88;74.05] | 75.48 [58.70;96.17] | 92.86 [73.20;113.71] | 113.87 [90.88;138.40] | 0.000 |
| TyG-CVAI | 706.41 [497.80;962.03] | 446.05 [294.57;596.39] | 636.93 [492.65;812.20] | 803.93 [626.06;992.49] | 1036.48 [806.78;1285.87] | 0.000 |

Model 1, unadjusted; Model 2, adjusted for age, gender, residence, BMI, UA, TC, eGFR, HbA1c, education level, marital status, smoking statues, and drinking statues;

**Table S3 Baseline Characteristics of participants according to quartiles of TyG-WWI**

|  | **[ALL]** | **Q1** | **Q2** | **Q3** | **Q4** | **p.overall** |
| --- | --- | --- | --- | --- | --- | --- |
| Variables | ***N=4541*** | ***N=1134*** | ***N=1131*** | ***N=1150*** | ***N=1126*** |  |
| Age,years | 56.00 [50.00;63.00] | 54.00 [48.00;61.00] | 56.00 [50.00;62.50] | 56.00 [51.00;63.00] | 58.00 [52.00;65.00] | <0.001 |
| Gender,n% |  |  |  |  |  | <0.001 |
| Male | 2151 (47.37%) | 721 (63.58%) | 624 (55.17%) | 483 (42.00%) | 323 (28.69%) |  |
| Female | 2390 (52.63%) | 413 (36.42%) | 507 (44.83%) | 667 (58.00%) | 803 (71.31%) |  |
| Marry,n% |  |  |  |  |  | 0.009 |
| Yes | 3923 (86.39%) | 998 (88.01%) | 991 (87.62%) | 993 (86.35%) | 941 (83.57%) |  |
| Education,n% |  |  |  |  |  | <0.001 |
| Below middle school | 3071 (67.63%) | 700 (61.73%) | 749 (66.22%) | 781 (67.91%) | 841 (74.69%) |  |
| Middle school and high school | 1419 (31.25%) | 418 (36.86%) | 369 (32.63%) | 354 (30.78%) | 278 (24.69%) |  |
| College or higher | 51 (1.12%) | 16 (1.41%) | 13 (1.15%) | 15 (1.30%) | 7 (0.62%) |  |
| Residence,n% |  |  |  |  |  | 0.009 |
| Urban | 1530 (33.69%) | 346 (30.51%) | 367 (32.45%) | 403 (35.04%) | 414 (36.77%) |  |
| Rural | 3011 (66.31%) | 788 (69.49%) | 764 (67.55%) | 747 (64.96%) | 712 (63.23%) |  |
| BMI (kg/m2) | 22.53 [20.41;24.82] | 21.22 [19.62;23.11] | 22.13 [20.15;24.09] | 23.09 [20.88;25.38] | 24.15 [21.69;26.48] | <0.001 |
| Drinking,n% |  |  |  |  |  | <0.001 |
| Never | 2652 (58.40%) | 558 (49.21%) | 626 (55.35%) | 712 (61.91%) | 756 (67.14%) |  |
| Current | 1575 (34.68%) | 503 (44.36%) | 413 (36.52%) | 348 (30.26%) | 311 (27.62%) |  |
| Former | 314 (6.91%) | 73 (6.44%) | 92 (8.13%) | 90 (7.83%) | 59 (5.24%) |  |
| Smoking,n% |  |  |  |  |  | <0.001 |
| Never | 2741 (60.36%) | 554 (48.85%) | 634 (56.06%) | 731 (63.57%) | 822 (73.00%) |  |
| Now | 1444 (31.80%) | 492 (43.39%) | 409 (36.16%) | 317 (27.57%) | 226 (20.07%) |  |
| Former | 356 (7.84%) | 88 (7.76%) | 88 (7.78%) | 102 (8.87%) | 78 (6.93%) |  |
| Diabetes,n%: |  |  |  |  |  | <0.001 |
| No | 3990 (87.87%) | 1085 (95.68%) | 1049 (92.75%) | 1017 (88.43%) | 839 (74.51%) |  |
| Yes | 551 (12.13%) | 49 (4.32%) | 82 (7.25%) | 133 (11.57%) | 287 (25.49%) |  |
| Dyslipidemia,n%: |  |  |  |  |  | <0.001 |
| No | 2614 (57.56%) | 913 (80.51%) | 764 (67.55%) | 607 (52.78%) | 330 (29.31%) |  |
| Yes | 1927 (42.44%) | 221 (19.49%) | 367 (32.45%) | 543 (47.22%) | 796 (70.69%) |  |
| Heart disease,n%: |  |  |  |  |  | 0.002 |
| No | 4117 (90.66%) | 1041 (91.80%) | 1049 (92.75%) | 1030 (89.57%) | 997 (88.54%) |  |
| Yes | 424 (9.34%) | 93 (8.20%) | 82 (7.25%) | 120 (10.43%) | 129 (11.46%) |  |
| Kidney disease,n% |  |  |  |  |  | 0.955 |
| No | 4152 (91.43%) | 1036 (91.36%) | 1038 (91.78%) | 1052 (91.48%) | 1026 (91.12%) |  |
| Yes | 389 (8.57%) | 98 (8.64%) | 93 (8.22%) | 98 (8.52%) | 100 (8.88%) |  |
| Stroke,n% |  |  |  |  |  | 0.124 |
| No | 4481 (98.68%) | 1118 (98.59%) | 1120 (99.03%) | 1139 (99.04%) | 1104 (98.05%) |  |
| Yes | 60 (1.32%) | 16 (1.41%) | 11 (0.97%) | 11 (0.96%) | 22 (1.95%) |  |
| Lung disease,n% |  |  |  |  |  | 0.770 |
| No | 4032 (88.79%) | 1014 (89.42%) | 998 (88.24%) | 1025 (89.13%) | 995 (88.37%) |  |
| Yes | 509 (11.21%) | 120 (10.58%) | 133 (11.76%) | 125 (10.87%) | 131 (11.63%) |  |
| Asthma,n% |  |  |  |  |  | 0.415 |
| No | 4343 (95.64%) | 1085 (95.68%) | 1089 (96.29%) | 1101 (95.74%) | 1068 (94.85%) |  |
| Yes | 198 (4.36%) | 49 (4.32%) | 42 (3.71%) | 49 (4.26%) | 58 (5.15%) |  |
| SBP(mmHg) | 118.33 [109.67;127.33] | 116.00 [108.00;125.00] | 117.67 [109.00;126.00] | 118.67 [110.33;128.25] | 121.00 [112.00;129.33] | <0.001 |
| DBP(mmHg) | 70.33 [64.33;76.67] | 69.33 [63.33;75.67] | 70.00 [64.00;76.50] | 71.00 [65.00;76.67] | 71.67 [65.33;78.00] | <0.001 |
| eGFR | 96.56 [86.90;104.03] | 98.53 [89.32;105.62] | 96.69 [87.92;104.07] | 96.30 [85.30;103.19] | 95.04 [84.69;102.73] | <0.001 |
| FBG (mg/dl) | 100.80 [93.42;109.98] | 96.12 [89.50;103.68] | 98.82 [92.16;106.74] | 101.61 [94.50;109.98] | 108.09 [98.86;123.44] | <0.001 |
| TG (mg/dl) | 96.46 [70.80;139.83] | 65.49 [52.22;81.42] | 85.85 [69.03;107.97] | 107.97 [86.73;140.71] | 162.40 [115.05;230.76] | 0.000 |
| Scr (mg/dl) | 0.75 [0.64;0.87] | 0.77 [0.67;0.88] | 0.76 [0.66;0.88] | 0.73 [0.63;0.86] | 0.72 [0.62;0.84] | <0.001 |
| UA (mg/dl) | 4.17 [3.49;4.95] | 4.17 [3.47;4.83] | 4.16 [3.49;4.95] | 4.15 [3.50;4.98] | 4.22 [3.51;5.04] | 0.349 |
| BUN (mg/dl) | 15.07 [12.49;18.07] | 15.63 [12.86;18.71] | 15.15 [12.49;18.18] | 14.93 [12.35;17.98] | 14.79 [12.36;17.48] | <0.001 |
| LDL-C (mg/dl) | 114.05 [93.17;135.70] | 106.12 [89.30;125.55] | 114.82 [94.33;133.38] | 117.53 [97.42;139.95] | 118.69 [93.94;142.66] | <0.001 |
| HDL-C (mg/dl) | 51.03 [41.75;61.47] | 56.83 [48.33;67.27] | 53.35 [44.07;62.63] | 49.10 [40.98;58.76] | 44.07 [35.57;53.74] | <0.001 |
| HbA1c (%) | 5.10 [4.90;5.40] | 5.10 [4.80;5.30] | 5.10 [4.80;5.30] | 5.20 [4.90;5.40] | 5.20 [5.00;5.60] | <0.001 |
| TC (mg/dl) | 188.66 [165.85;213.40] | 175.90 [157.83;201.03] | 185.95 [163.34;206.83] | 190.98 [168.94;214.95] | 202.58 [178.61;231.96] | <0.001 |
| TyG | 8.50 [8.15;8.91] | 8.06 [7.81;8.29] | 8.38 [8.13;8.60] | 8.63 [8.40;8.91] | 9.11 [8.75;9.57] | 0.000 |
| BRI | 3.74 [3.01;4.67] | 2.77 [2.31;3.23] | 3.47 [2.97;4.00] | 4.07 [3.51;4.82] | 4.98 [4.27;5.72] | 0.000 |
| TyG-BRI | 31.87 [25.11;40.61] | 22.14 [18.58;25.77] | 29.11 [25.22;33.31] | 35.49 [30.50;41.08] | 45.46 [38.85;52.61] | 0.000 |
| ABSI | 0.08 [0.08;0.09] | 0.08 [0.08;0.08] | 0.08 [0.08;0.08] | 0.08 [0.08;0.09] | 0.09 [0.08;0.09] | 0.000 |
| TyG-ABSI | 0.70 [0.66;0.75] | 0.63 [0.60;0.65] | 0.68 [0.66;0.70] | 0.72 [0.70;0.74] | 0.79 [0.76;0.82] | 0.000 |
| WWI | 10.98 [10.45;11.54] | 10.21 [9.83;10.50] | 10.78 [10.46;11.10] | 11.21 [10.92;11.60] | 11.81 [11.38;12.30] | 0.000 |
| TyG-WWI | 93.62 [86.88;100.95] | 82.51 [78.94;84.90] | 90.26 [88.65;92.03] | 96.95 [95.16;99.10] | 106.72 [103.47;112.03] | 0.000 |
| CVAI | 83.28 [60.09;110.18] | 51.80 [34.45;67.77] | 75.09 [60.28;92.12] | 93.02 [74.97;112.90] | 118.11 [96.67;138.98] | 0.000 |
| TyG-CVAI | 706.41 [497.80;962.03] | 414.21 [273.62;547.05] | 627.67 [504.80;781.11] | 802.54 [654.57;972.20] | 1072.20 [870.95;1293.87] | 0.000 |

**Table S4 Baseline Characteristics of participants according to quartiles of TyG-CVAI**

|  | **[ALL]** | **Q1** | **Q2** | **Q3** | **Q4** | **p.overall** |
| --- | --- | --- | --- | --- | --- | --- |
| Variables | ***N=4541*** | ***N=1137*** | ***N=1138*** | ***N=1141*** | ***N=1125*** |  |
| Age,years | 56.00 [50.00;63.00] | 55.00 [48.00;62.00] | 56.00 [50.00;61.00] | 57.00 [51.00;63.00] | 58.00 [52.00;64.00] | <0.001 |
| Gender,n% |  |  |  |  |  | <0.001 |
| Male | 2151 (47.37%) | 680 (59.81%) | 532 (46.75%) | 429 (37.60%) | 510 (45.33%) |  |
| Female | 2390 (52.63%) | 457 (40.19%) | 606 (53.25%) | 712 (62.40%) | 615 (54.67%) |  |
| Marry,n% |  |  |  |  |  | 0.679 |
| Yes | 3923 (86.39%) | 987 (86.81%) | 991 (87.08%) | 975 (85.45%) | 970 (86.22%) |  |
| Education,n% |  |  |  |  |  | 0.473 |
| Below middle school | 3071 (67.63%) | 766 (67.37%) | 777 (68.28%) | 792 (69.41%) | 736 (65.42%) |  |
| Middle school and high school | 1419 (31.25%) | 359 (31.57%) | 351 (30.84%) | 336 (29.45%) | 373 (33.16%) |  |
| College or higher | 51 (1.12%) | 12 (1.06%) | 10 (0.88%) | 13 (1.14%) | 16 (1.42%) |  |
| Residence,n% |  |  |  |  |  | <0.001 |
| Urban | 1530 (33.69%) | 312 (27.44%) | 326 (28.65%) | 385 (33.74%) | 507 (45.07%) |  |
| Rural | 3011 (66.31%) | 825 (72.56%) | 812 (71.35%) | 756 (66.26%) | 618 (54.93%) |  |
| BMI (kg/m2) | 22.53 [20.41;24.82] | 19.82 [18.64;21.25] | 21.70 [20.31;23.08] | 23.32 [21.71;24.78] | 25.92 [24.16;27.88] | 0.000 |
| Drinking,n% |  |  |  |  |  | <0.001 |
| Never | 2652 (58.40%) | 588 (51.72%) | 661 (58.08%) | 728 (63.80%) | 675 (60.00%) |  |
| Current | 1575 (34.68%) | 475 (41.78%) | 392 (34.45%) | 332 (29.10%) | 376 (33.42%) |  |
| Former | 314 (6.91%) | 74 (6.51%) | 85 (7.47%) | 81 (7.10%) | 74 (6.58%) |  |
| Smoking,n% |  |  |  |  |  | <0.001 |
| Never | 2741 (60.36%) | 569 (50.04%) | 688 (60.46%) | 780 (68.36%) | 704 (62.58%) |  |
| Now | 1444 (31.80%) | 495 (43.54%) | 378 (33.22%) | 281 (24.63%) | 290 (25.78%) |  |
| Former | 356 (7.84%) | 73 (6.42%) | 72 (6.33%) | 80 (7.01%) | 131 (11.64%) |  |
| Diabetes,n%: |  |  |  |  |  | <0.001 |
| No | 3990 (87.87%) | 1056 (92.88%) | 1048 (92.09%) | 1001 (87.73%) | 885 (78.67%) |  |
| Yes | 551 (12.13%) | 81 (7.12%) | 90 (7.91%) | 140 (12.27%) | 240 (21.33%) |  |
| Dyslipidemia,n%: |  |  |  |  |  | <0.001 |
| No | 2614 (57.56%) | 922 (81.09%) | 772 (67.84%) | 616 (53.99%) | 304 (27.02%) |  |
| Yes | 1927 (42.44%) | 215 (18.91%) | 366 (32.16%) | 525 (46.01%) | 821 (72.98%) |  |
| Heart disease,n%: |  |  |  |  |  | <0.001 |
| No | 4117 (90.66%) | 1051 (92.44%) | 1056 (92.79%) | 1039 (91.06%) | 971 (86.31%) |  |
| Yes | 424 (9.34%) | 86 (7.56%) | 82 (7.21%) | 102 (8.94%) | 154 (13.69%) |  |
| Kidney disease,n% |  |  |  |  |  | 0.150 |
| No | 4152 (91.43%) | 1032 (90.77%) | 1059 (93.06%) | 1035 (90.71%) | 1026 (91.20%) |  |
| Yes | 389 (8.57%) | 105 (9.23%) | 79 (6.94%) | 106 (9.29%) | 99 (8.80%) |  |
| Stroke,n% |  |  |  |  |  | 0.009 |
| No | 4481 (98.68%) | 1122 (98.68%) | 1131 (99.38%) | 1128 (98.86%) | 1100 (97.78%) |  |
| Yes | 60 (1.32%) | 15 (1.32%) | 7 (0.62%) | 13 (1.14%) | 25 (2.22%) |  |
| Lung disease,n% |  |  |  |  |  | 0.074 |
| No | 4032 (88.79%) | 989 (86.98%) | 1013 (89.02%) | 1032 (90.45%) | 998 (88.71%) |  |
| Yes | 509 (11.21%) | 148 (13.02%) | 125 (10.98%) | 109 (9.55%) | 127 (11.29%) |  |
| Asthma,n% |  |  |  |  |  | 0.565 |
| No | 4343 (95.64%) | 1083 (95.25%) | 1091 (95.87%) | 1098 (96.23%) | 1071 (95.20%) |  |
| Yes | 198 (4.36%) | 54 (4.75%) | 47 (4.13%) | 43 (3.77%) | 54 (4.80%) |  |
| SBP(mmHg) | 118.33 [109.67;127.33] | 114.33 [106.67;124.33] | 117.67 [109.33;126.67] | 118.00 [110.33;127.00] | 122.67 [114.33;130.33] | <0.001 |
| DBP(mmHg) | 70.33 [64.33;76.67] | 68.33 [62.33;75.00] | 70.00 [64.00;76.33] | 70.67 [64.67;76.33] | 72.67 [66.67;78.67] | <0.001 |
| eGFR | 96.56 [86.90;104.03] | 98.47 [89.93;105.51] | 98.04 [88.33;104.66] | 95.84 [85.93;103.14] | 94.13 [82.86;102.09] | <0.001 |
| FBG (mg/dl) | 100.80 [93.42;109.98] | 97.20 [90.54;105.30] | 98.46 [91.80;106.33] | 101.34 [94.68;109.62] | 106.56 [97.20;118.98] | <0.001 |
| TG (mg/dl) | 96.46 [70.80;139.83] | 69.92 [54.87;89.39] | 85.85 [67.26;111.51] | 106.20 [81.42;143.37] | 150.45 [110.62;216.82] | <0.001 |
| Scr (mg/dl) | 0.75 [0.64;0.87] | 0.76 [0.64;0.88] | 0.73 [0.63;0.85] | 0.73 [0.63;0.85] | 0.76 [0.66;0.88] | <0.001 |
| UA (mg/dl) | 4.17 [3.49;4.95] | 4.13 [3.46;4.87] | 4.04 [3.35;4.78] | 4.08 [3.43;4.81] | 4.47 [3.75;5.30] | <0.001 |
| BUN (mg/dl) | 15.07 [12.49;18.07] | 15.63 [12.83;18.75] | 14.96 [12.46;17.89] | 14.90 [12.35;17.79] | 14.87 [12.49;17.65] | <0.001 |
| LDL-C (mg/dl) | 114.05 [93.17;135.70] | 107.47 [88.14;126.80] | 112.11 [92.78;133.76] | 117.53 [98.20;139.95] | 118.30 [94.52;140.34] | <0.001 |
| HDL-C (mg/dl) | 51.03 [41.75;61.47] | 60.31 [51.03;71.13] | 54.51 [45.62;63.40] | 49.48 [41.75;57.22] | 41.37 [34.41;48.33] | <0.001 |
| HbA1c (%) | 5.10 [4.90;5.40] | 5.10 [4.90;5.30] | 5.10 [4.80;5.30] | 5.20 [4.90;5.40] | 5.20 [5.00;5.50] | <0.001 |
| TC (mg/dl) | 188.66 [165.85;213.40] | 182.09 [160.83;206.44] | 185.95 [162.76;209.44] | 189.43 [167.40;214.56] | 196.39 [173.58;221.91] | <0.001 |
| TyG | 8.50 [8.15;8.91] | 8.14 [7.88;8.41] | 8.36 [8.09;8.64] | 8.59 [8.35;8.93] | 9.02 [8.66;9.48] | <0.001 |
| BRI | 3.74 [3.01;4.67] | 2.66 [2.27;3.09] | 3.42 [3.03;3.87] | 4.09 [3.62;4.70] | 5.13 [4.50;5.85] | 0.000 |
| TyG-BRI | 31.87 [25.11;40.61] | 21.69 [18.55;25.04] | 28.74 [25.40;32.10] | 35.61 [31.54;40.32] | 46.42 [40.44;53.29] | 0.000 |
| ABSI | 0.08 [0.08;0.09] | 0.08 [0.08;0.08] | 0.08 [0.08;0.08] | 0.08 [0.08;0.09] | 0.08 [0.08;0.09] | <0.001 |
| TyG-ABSI | 0.70 [0.66;0.75] | 0.65 [0.61;0.68] | 0.68 [0.65;0.71] | 0.72 [0.68;0.75] | 0.76 [0.73;0.81] | 0.000 |
| WWI | 10.98 [10.45;11.54] | 10.37 [9.95;10.79] | 10.80 [10.40;11.26] | 11.17 [10.76;11.66] | 11.51 [11.06;11.98] | <0.001 |
| TyG-WWI | 93.62 [86.88;100.95] | 84.35 [79.72;88.80] | 90.53 [86.79;94.85] | 96.55 [92.28;101.43] | 103.76 [98.74;111.07] | 0.000 |
| CVAI | 83.28 [60.09;110.18] | 43.97 [31.92;52.57] | 72.21 [66.48;78.05] | 95.35 [88.97;102.11] | 130.28 [118.75;146.94] | 0.000 |
| TyG-CVAI | 706.41 [497.80;962.03] | 364.92 [255.91;433.31] | 607.81 [552.22;656.54] | 824.90 [763.50;889.15] | 1171.63 [1049.12;1350.10] | 0.000 |
