# Supplementary material for: Associations between novel triglyceride-glucose-related indices and the incidence of hypertension among Chinese middle-aged and elderly adults: a nationwide prospective cohort study
Source: Cardiovasc Diabetol Endocrinol Rep. 2025 Dec 29;11:50. doi: 10.1186/s40842-025-00255-3 (PMC12746625; doi:10.1186/s40842-025-00255-3)
Supplement: Supplementary file 2 — Supplementary Material 2 [file 40842_2025_255_MOESM2_ESM.docx]

|  | TyG-BRI | TyG-ABSI | TyG-WWI | TyG-CVAI |
| --- | --- | --- | --- | --- |
| TyG-related indice | 1.32 | 1.19 | 1.23 | 1.50 |
| Rural | 1.10 | 1.10 | 1.10 | 1.10 |
| Drinking ever | 1.34 | 1.34 | 1.34 | 1.34 |
| Drinking never | 1.11 | 1.11 | 1.11 | 1.11 |
| Education level  Middle school and high school | 1.11 | 1.11 | 1.12 | 1.11 |
| Education level  College or higher | 1.04 | 1.04 | 1.04 | 1.04 |
| Marry | 1.02 | 1.02 | 1.02 | 1.02 |
| BMI | 1.14 | 1.04 | 1.03 | 1.16 |
| Smoking ever | 1.39 | 1.34 | 1.36 | 1.36 |
| Smoking never | 1.17 | 1.17 | 1.17 | 1.17 |
| HbA1c | 1.32 | 1.32 | 1.32 | 1.32 |
| UA | 1.35 | 1.35 | 1.34 | 1.35 |
| TC | 1.14 | 1.14 | 1.15 | 1.13 |
| EGFR | 1.27 | 1.27 | 1.27 | 1.28 |
| Kidney disease | 1.06 | 1.06 | 1.06 | 1.07 |
| Stroke | 1.01 | 1.01 | 1.01 | 1.01 |
| Diabetes | 1.31 | 1.32 | 1.32 | 1.31 |
| Dyslipidemia | 1.20 | 1.22 | 1.22 | 1.33 |
| Cardiovascular disease | 1.05 | 1.05 | 1.05 | 1.05 |
| Lung disease | 1.24 | 1.24 | 1.24 | 1.24 |
| Asthma | 1.24 | 1.24 | 1.24 | 1.24 |

Table S1 Variance Inflation Factors of Covariates in Cox Model 3.(fully adjusted model)

**Figure S1. Calibration plot using K-M method (fully adjusted Cox Model) of four TyG-related indices**


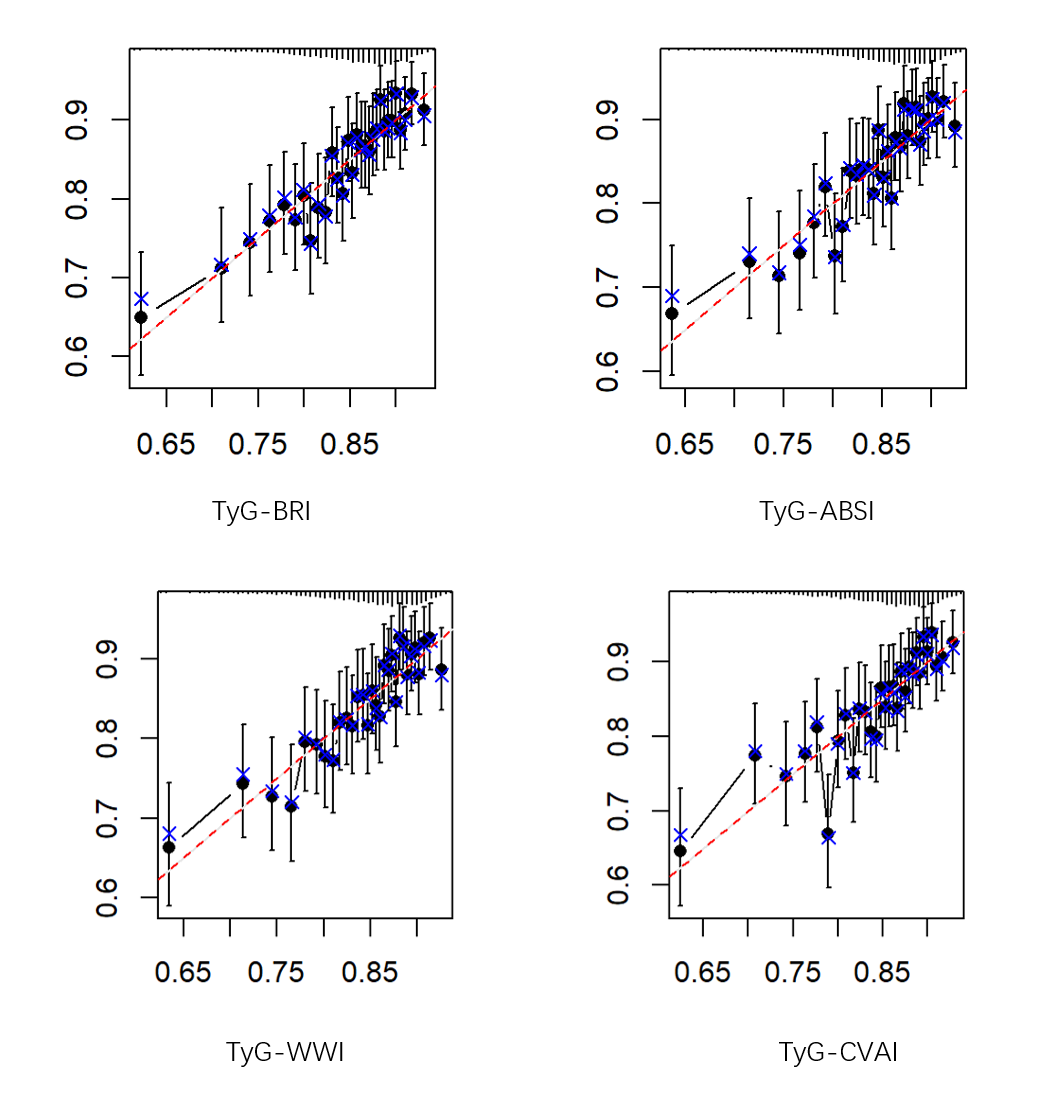


**Figure S2. Brier score of four TyG-related indices at median follow-up (fully adjusted cox model)**


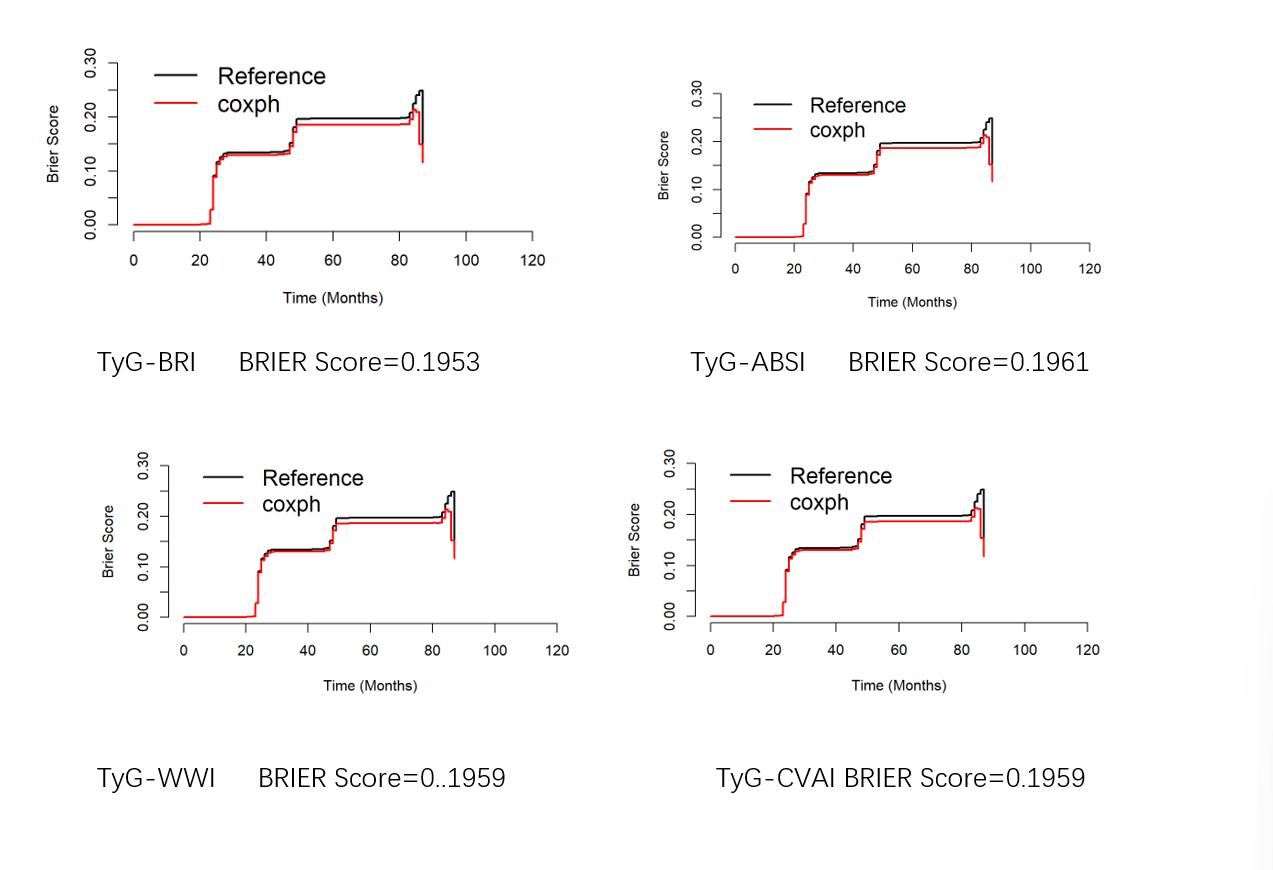


**Figure S3 Grønnesby–Borgan test for fully adjusted Cox Model**


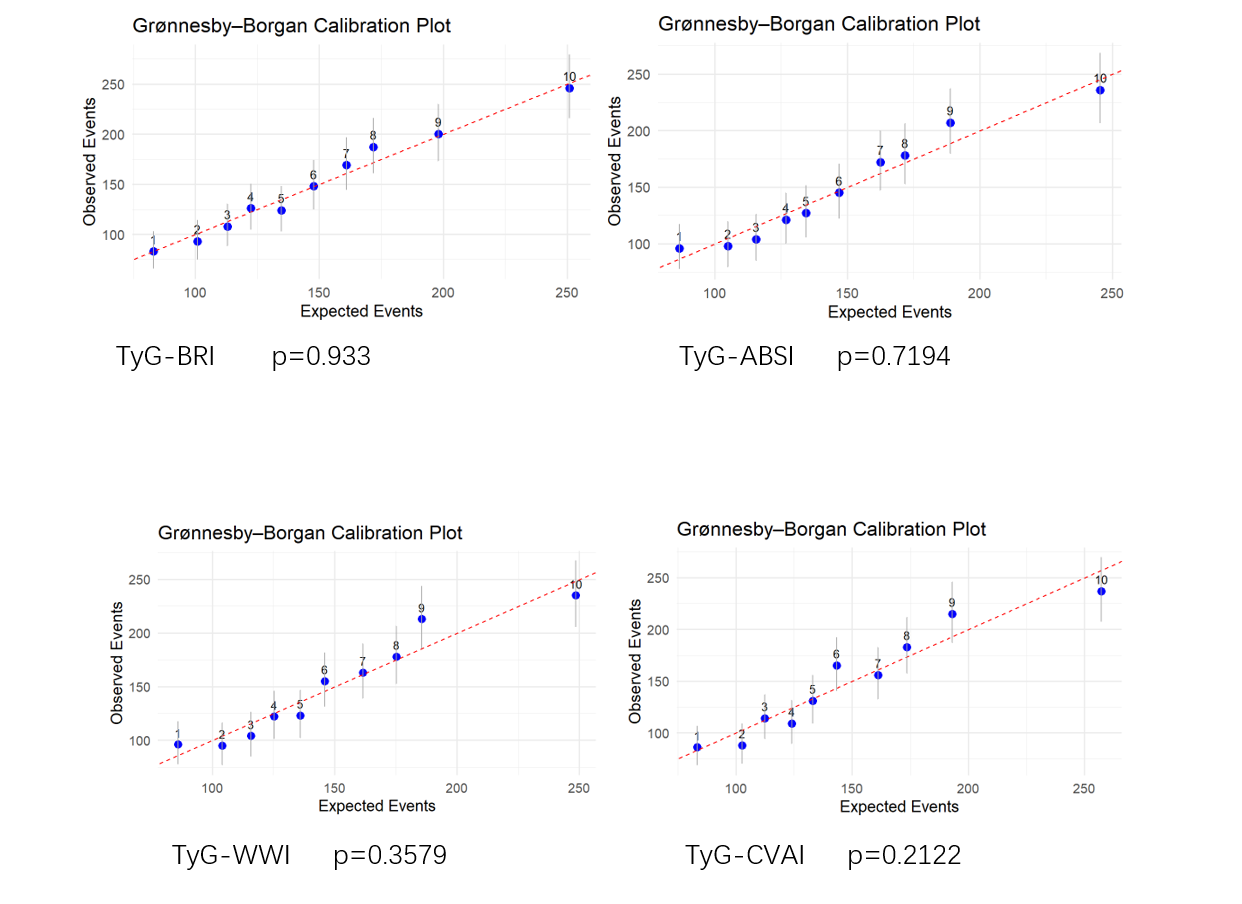


**Table S2 Cox-ZPH Test for TyG-related indices of fully adjustd Cox Model**

|  | TyG-BRI  p.value | TyG-ABSI  p.value | TyGWWI  p.value | TyG-CVAI  p.value |
| --- | --- | --- | --- | --- |
| TyG-related  indices | 0.33 | 0.13 | 0.15 | 0.22 |
| Age | 0.40 | 0.39 | 0.39 | 0.36 |
| Gender | 0.41 | 0.45 | 0.45 | 0.39 |
| Residance | 0.40 | 0.37 | 0.38 | 0.39 |
| Drinking | 0.98 | 0.98 | 0.98 | 0.98 |
| Education level | 0.32 | 0.34 | 0.34 | 0.33 |
| Marry | 0.65 | 0.70 | 0.68 | 0.69 |
| BMI | 0.87 | 0.80 | 0.81 | 0.88 |
| Smoking | 0.56 | 0.59 | 0.58 | 0.54 |
| HbA1c | 0.83 | 0.90 | 0.89 | 0.86 |
| UA | 0.45 | 0.49 | 0.48 | 0.43 |
| TC | 0.45 | 0.47 | 0.47 | 0.45 |
| EGFR | 0.79 | 0.78 | 0.78 | 0.75 |
| Kidney disease | 0.82 | 0.80 | 0.80 | 0.79 |
| Stroke | 0.41 | 0.39 | 0.39 | 0.40 |
| Diabetes | 0.71 | 0.70 | 0.69 | 0.72 |
| Dyslipidemia | 0.14 | 0.15 | 0.15 | 0.13 |
| Cardiovascular disease | 0.58 | 0.59 | 0.58 | 0.57 |
| Lung disease | 0.54 | 0.55 | 0.55 | 0.57 |
| Asthma | 0.61 | 0.66 | 0.65 | 0.60 |
| Global | 0.97 | 0.95 | 0.96 | 0.94 |
