# Supplementary material for: Associations between novel triglyceride-glucose-related indices and the incidence of hypertension among Chinese middle-aged and elderly adults: a nationwide prospective cohort study
Source: Cardiovasc Diabetol Endocrinol Rep. 2025 Dec 29;11:50. doi: 10.1186/s40842-025-00255-3 (PMC12746625; doi:10.1186/s40842-025-00255-3)

**Table S1 P-value of Delong‘s test among TyG index, base model, TyG-related indices**

|  | TyG-BRI | TyG-ABSI | TyG-WWI | TyG-CVAI |
| --- | --- | --- | --- | --- |
| T=24 months |  |  |  |  |
| TyG index | 0.07 | 0.003* | 0.003* | 0.32 |
| base model | 0.05* | 0.004* | 0.005* | 0.17 |
| TyG-BRI |  | 0.32 | 0.40 | 0.72 |
| TyG-ABSI | 0.32 |  | 0.20 | 0.67 |
| TyG-WWI | 0.40 | 0.20 |  | 0.85 |
| TyG-CVAI | 0.72 | 0.67 | 0.85 |  |
| T=48 months |  |  |  |  |
| TyG index | 0.04* | 0.15 | 0.07 | 0.08 |
| base model | 0.03* | 0.11 | 0.07 | 0.09 |
| TyG-BRI |  | 0.09 | 0.14 | 0.79 |
| TyG-ABSI | 0.09 |  | 0.08 | 0.33 |
| TyG-WWI | 0.14 | 0.08 |  | 0.56 |
| TyG-CVAI | 0.79 | 0.33 | 0.56 |  |
| T=60 months |  |  |  |  |
| TyG index | 0.04* | 0.39 | 0.19 | 0.03^*^ |
| base model | 0.02* | 0.12 | 0.07 | 0.02^*^ |
| TyG-BRI |  | 0.05* | 0.07 | 1.00 |
| TyG-ABSI | 0.05* |  | 0.09 | 0.08 |
| TyG-WWI | 0.07 | 0.09 |  | 0.17 |
| TyG-CVAI | 1.00 | 0.08 | 0.17 |  |
| T=median follow-up(83 months) |  |  |  |  |
| TyG index | 0.14 | 0.31 | 0.18 | 0.19 |
| base model | 0.09 | 0.17 | 0.12 | 0.16 |
| TyG-BRI |  | 0.25 | 0.34 | 0.91 |
| TyG-ABSI | 0.25 |  | 0.17 | 0.52 |
| TyG-WWI | 0.34 | 0.17 |  | 0.75 |
| TyG-CVAI | 0.91 | 0.52 | 0.75 |  |

**Table S2 Comparison of predictive performance between TyG index, TyG-related indices using NRI at four follow-up time points.**

| **Indicator** | **NRI Component** | **24 Months** | **48 Months** | **60 Months** | **83 Months** |
| --- | --- | --- | --- | --- | --- |
| TyG-ABSI | NRI | 0.071 (-0.075 - 0.165) | 0.098 (-0.027 - 0.168) | 0.111 (-0.008 - 0.190) | 0.106 (-0.028 - 0.165) |
|  | NRI- (Non-events) | -0.077 (-0.132 - 0.012) | -0.072 (-0.115 - 0.010) | -0.070 (-0.110 - 0.028) | -0.073 (-0.116 - 0.031) |
|  | NRI+ (Events) | 0.148 (-0.034 - 0.247) | 0.170 (0.034 - 0.206) | 0.181 (0.059 - 0.211) | 0.179 (0.051 - 0.196) |
| TyG-BRI | NRI | 0.110 (0.003 - 0.226) | 0.150 (0.022 - 0.214) | 0.153 (0.025 - 0.229) | 0.142 (0.034 - 0.192) |
|  | NRI- (Non-events) | 0.115 (0.004 - 0.159) | 0.124 (-0.024 - 0.166) | 0.125 (-0.025 - 0.168) | 0.123 (-0.012 - 0.157) |
|  | NRI+ (Events) | -0.005 (-0.077 - 0.154) | 0.026 (-0.029 - 0.095) | 0.028 (-0.020 - 0.112) | 0.018 (-0.029 - 0.113) |
| TyG-CVAI | NRI | 0.103 (-0.047 - 0.179) | 0.147 (0.028 - 0.204) | 0.165 (0.046 - 0.245) | 0.147 (0.035 - 0.224) |
|  | NRI- (Non-events) | 0.100 (-0.028 - 0.129) | 0.117 (-0.013 - 0.145) | 0.124 (-0.011 - 0.164) | 0.121 (-0.015 - 0.156) |
|  | NRI+ (Events) | 0.003 (-0.078 - 0.124) | 0.029 (-0.027 - 0.123) | 0.041 (0.000 - 0.131) | 0.026 (-0.012 - 0.111) |
| TyG-WWI | NRI | 0.087 (-0.029 - 0.159) | 0.102 (0.014 - 0.172) | 0.112 (0.008 - 0.166) | 0.115 (0.012 - 0.175) |
|  | NRI- (Non-events) | -0.038 (-0.093 - 0.018) | -0.036 (-0.094 - 0.028) | -0.036 (-0.097 - 0.035) | -0.033 (-0.105 - 0.046) |
|  | NRI+ (Events) | 0.125 (-0.003 - 0.233) | 0.138 (0.041 - 0.204) | 0.148 (0.039 - 0.181) | 0.148 (0.050 - 0.186) |
| *Data are presented as point estimate (95% confidence interval). NRI+ indicates improvement in reclassification among events, and NRI- indicates improvement among non-events.* | | | | | |

**Table S3 Comparison of predictive performance between TyG index and base model using NRI at four follow-up time points.**

| **Indicator** | **NRI Component** | **24 Months** | **48 Months** | **60 Months** | **83 Months** |
| --- | --- | --- | --- | --- | --- |
| TyG-ABSI | NRI | -0.010 (-0.100 - 0.105) | 0.085 (0.006 - 0.144) | 0.101 (0.032 - 0.185) | 0.102 (0.003 - 0.173) |
|  | NRI- (Non-events) | -0.039 (-0.067 - 0.014) | -0.024 (-0.057 - 0.041) | -0.020 (-0.057 - 0.037) | -0.019 (-0.058 - 0.032) |
|  | NRI+ (Events) | 0.028 (-0.064 - 0.124) | 0.109 (0.018 - 0.132) | 0.120 (0.046 - 0.152) | 0.121 (0.045 - 0.165) |
| TyG-BRI | NRI | 0.114 (-0.024 - 0.181) | 0.163 (0.033 - 0.225) | 0.177 (0.051 - 0.228) | 0.157 (0.084 - 0.225) |
|  | NRI- (Non-events) | 0.128 (0.032 - 0.170) | 0.141 (0.019 - 0.169) | 0.147 (0.050 - 0.185) | 0.143 (0.045 - 0.177) |
|  | NRI+ (Events) | -0.014 (-0.102 - 0.090) | 0.022 (-0.041 - 0.080) | 0.030 (-0.017 - 0.078) | 0.014 (-0.014 - 0.079) |
| TyG-CVAI | NRI | 0.065 (-0.099 - 0.149) | 0.123 (0.019 - 0.190) | 0.149 (0.063 - 0.216) | 0.131 (0.057 - 0.225) |
|  | NRI- (Non-events) | 0.098 (0.001 - 0.138) | 0.114 (0.010 - 0.148) | 0.123 (0.033 - 0.156) | 0.118 (0.019 - 0.160) |
|  | NRI+ (Events) | -0.032 (-0.122 - 0.043) | 0.008 (-0.047 - 0.081) | 0.026 (-0.010 - 0.100) | 0.014 (-0.024 - 0.084) |
| TyG-WWI | NRI | 0.026 (-0.087 - 0.109) | 0.098 (-0.001 - 0.168) | 0.112 (0.029 - 0.165) | 0.112 (0.057 - 0.179) |
|  | NRI- (Non-events) | -0.002 (-0.045 - 0.037) | 0.008 (-0.051 - 0.060) | 0.012 (-0.030 - 0.052) | 0.011 (-0.038 - 0.055) |
|  | NRI+ (Events) | 0.029 (-0.086 - 0.108) | 0.090 (0.022 - 0.139) | 0.101 (0.025 - 0.141) | 0.102 (0.049 - 0.146) |
| *Data are presented as point estimate (95% confidence interval). NRI+ indicates improvement in reclassification among events, and NRI- indicates improvement among non-events.* | | | | | |

**Table S4 Comparison of predictive performance between TyG index, TyG-related indices using IDI at four follow-up time points.**

| **Follow-up Time** | **TyG-WWI** | **TyG-BRI** | **TyG-ABSI** | **TyG-CVAI** |
| --- | --- | --- | --- | --- |
| 24 months | 0.00004 (-0.00013 - 0.00016) | 0.00019 (0.00001 - 0.00049) | 0.00004 (-0.00013 - 0.00017) | 0.00024 (0.00001 - 0.00061) |
| 48 months | 0.00009 (-0.00032 - 0.00043) | 0.00052 (0.00003 - 0.00152) | 0.00010 (-0.00034 - 0.00047) | 0.00064 (0.00002 - 0.00186) |
| 60 months | 0.00010 (-0.00037 - 0.00049) | 0.00060 (0.00004 - 0.00171) | 0.00012 (-0.00039 - 0.00053) | 0.00076 (0.00002 - 0.00215) |
| 83 months | 0.00012 (-0.00039 - 0.00054) | 0.00065 (0.00004 - 0.00185) | 0.00013 (-0.00044 - 0.00058) | 0.00082 (0.00003 - 0.00228) |
|  | | | | |

**Table S5 Comparison of predictive performance between TyG index and base model using IDI at four follow-up time points.**

| **Follow-up Time** | **TyG-WWI** | **TyG-BRI** | **TyG-ABSI** | **TyG-CVAI** |
| --- | --- | --- | --- | --- |
| 24 months | -0.00011 (-0.00033 - -0.00002) | 0.00005 (-0.00001 - 0.00016) | -0.00010 (-0.00034 - -0.00002) | 0.00010 (0.00001 - 0.00030) |
| 48 months | -0.00029 (-0.00066 - -0.00005) | 0.00013 (0.00000 - 0.00054) | -0.00028 (-0.00067 - -0.00006) | 0.00025 (0.00004 - 0.00080) |
| 60 months | -0.00034 (-0.00073 - -0.00006) | 0.00015 (0.00000 - 0.00063) | -0.00033 (-0.00074 - -0.00007) | 0.00031 (0.00005 - 0.00095) |
| 83 months | -0.00038 (-0.00079 - -0.00006) | 0.00016 (-0.00001 - 0.00065) | -0.00036 (-0.00080 - -0.00008) | 0.00032 (0.00006 - 0.00099) |
|  | | | | |

|  |
| --- |

**Figure S1 Time-dependent AUCs for TyG-related indices (Model 3)**


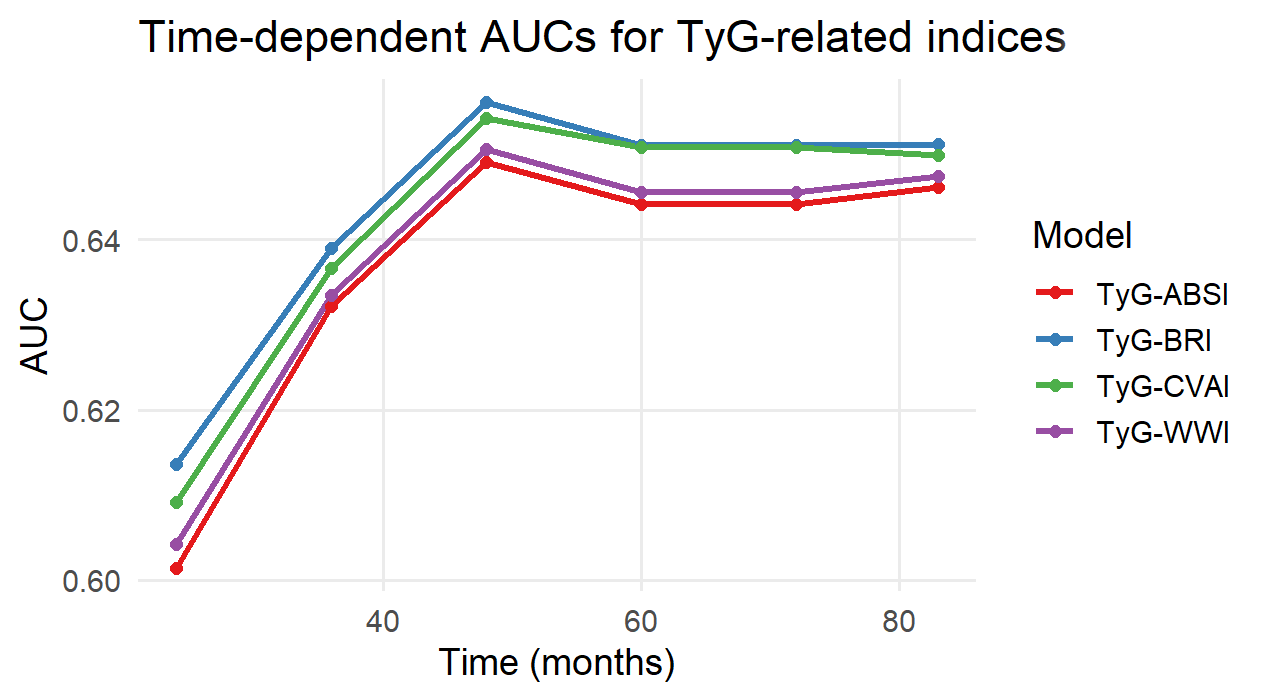

Supplement: Supplementary file 3 — Supplementary Material 3 [file 40842_2025_255_MOESM3_ESM.docx]
