# Supplementary material for: Associations between novel triglyceride-glucose-related indices and the incidence of hypertension among Chinese middle-aged and elderly adults: a nationwide prospective cohort study
Source: Cardiovasc Diabetol Endocrinol Rep. 2025 Dec 29;11:50. doi: 10.1186/s40842-025-00255-3 (PMC12746625; doi:10.1186/s40842-025-00255-3)
Supplement: Supplementary file 4 — Supplementary Material 4 [file 40842_2025_255_MOESM4_ESM.docx]

**Table S1 Cox regression analysis of TyG-related indices and new-onset hypertension risk after multiple imputation.**

|  | Model I | P-valve | Model II | P-valve | Model III | P-valve |
| --- | --- | --- | --- | --- | --- | --- |
|  | HR (95% CI) |  | HR (95% CI) |  | HR (95% CI) |  |
| TyG-BRI  (per 1 SD) | 1.25  (1.19-1.32) | <0.001* | 1.23  (1.17-1.31) | <0.001* | 1.23  (1.17-1.31) | <0.001* |
| TyG-BRI  Quartile |  |  |  |  |  |  |
| Q1 | Ref |  | Ref |  | Ref |  |
| Q2 | 1.17  (1.00-1.36) | 0.051 | 1.18  (1.01-1.38) | 0.040* | 1.18  (1.01-1.38) | 0.041* |
| Q3 | 1.39  (1.20-1.62) | <0.001* | 1.40  (1.20-1.64) | <0.001* | 1.39  (1.19-1.63) | <0.001* |
| Q4 | 1.71  (1.48-1.97) | <0.001* | 1.68  (1.43-1.97) | <0.001* | 1.64  (1.36-1.93) | <0.001* |
| TyG-ABSI  (per 1 SD) | 1.23  (1.16-1.30) | <0.001* | 1.12  (1.06 -1.19) | <0.001* | 1.10  (1.04-1.17) | 0.016* |
| TyG-ABSI  Quartile |  |  |  |  |  |  |
| Q1 | Ref |  | Ref |  | Ref |  |
| Q2 | 1.37  (1.17- 1.60) | <0.001* | 1.30  (1.11-1.52) | 0.001* | 1.29  (1.10-1.50) | 0.002* |
| Q3 | 1.55  (1.34- 1.81) | <0.001* | 1.40  (1.20-1.64) | <0.001* | 1.37  (1.18-1.61) | <0.001* |
| Q4 | 1.84  (1.59- 2.13) | <0.001* | 1.45  (1.24-1.70) | <0.001* | 1.38  (1.17 -1.64) | <0.001* |
| TyG-WWI  (per 1 SD) | 1.24  (1.18-1.32) | <0.001* | 1.15  (1.08-1.22) | <0.001* | 1.13  (1.06-1.20) | <0.001* |
| TyG-WWI  Quartile |  |  |  |  |  |  |
| Q1 | Ref |  | Ref |  | Ref |  |
| Q2 | 1.50  (1.29-1.75) | <0.001* | 1.46  (1.25-1.71) | <0.001* | 1.46  (1.25-1.70) | <0.001* |
| Q3 | 1.55  (1.33-1.81) | <0.001* | 1.45  (1.23-1.70) | <0.001* | 1.33  (1.22-1.68) | <0.001* |
| Q4 | 1.90  (1.64-2.21 ) | <0.001* | 1.61  (1.37-1.90) | <0.001* | 1.55  (1.30-1.84) | <0.001* |
| TyG-CVAI  (per 1 SD) | 1.3  (1.24-1.36) | <0.001* | 1.22  (1.16-1.29) | <0.001* | 1.22  (1.15-1.30) | <0.001* |
| TyG-CVAI Quartile |  |  |  |  |  |  |
| Q1 | Ref |  | Ref |  | Ref |  |
| Q2 | 1.01  (0.86-1.18) | 0.898 | 1.01  (0.88-1.18) | 0.974 | 1.00  (0.86-1.18) | 0.963 |
| Q3 | 1.34  (1.15-1.55) | <0.001* | 1.27  (1.09 -1.48) | 0.003* | 1.27  (1.09-1.48) | 0.003* |
| Q4 | 1.85  (1.61-2.13) | <0.001* | 1.61  (1.38-1.88) | <0.001* | 1.60  (1.36-1.89) | <0.001* |

**Table S2 Cox regression analysis of TyG-related indices and new-onset hypertension risk with retention of extreme values.**

|  | Model I | P-valve | Model II | P-valve | Model III | P-valve |
| --- | --- | --- | --- | --- | --- | --- |
|  | HR (95% CI) |  | HR (95% CI) |  | HR (95% CI) |  |
| TyG-BRI  (per 1 SD) | 1.04  (1.02-1.06) | <0.001* | 1.21  (1.08-1.36) | 0.001* | 1.17  (1.04-1.31) | 0.007* |
| TyG-BRI  Quartile |  |  |  |  |  |  |
| Q1 | Ref |  | Ref |  | Ref |  |
| Q2 | 1.14  (0.98-1.34) | 0.914 | 1.15  (0.99-1.35) | 0.074 | 1.15  (1.01-1.39) | 0.079 |
| Q3 | 1.36  (1.17-1.59) | <0.001* | 1.37  (1.18-1.61) | <0.001* | 1.26  (1.19-1.64) | <0.001* |
| Q4 | 1.66  (1.44-1.93) | <0.001* | 1.62  (1.38-1.91) | 0.001* | 1.58  (1.08-1.60) | <0.001* |
| TyG-ABSI  (per 1 SD) | 1.09  (1.06-1.11) | <0.001* | 1.05  (1.01 -1.09) | 0.010* | 1.04  (1.00-1.09) | 0.055 |
| TyG-ABSI  Quartile |  |  |  |  |  |  |
| Q1 | Ref |  | Ref |  | Ref |  |
| Q2 | 1.38  (1.18- 1.61) | <0.001* | 1.31  (1.12-1.53) | <0.001* | 1.29  (1.11-1.52) | 0.001* |
| Q3 | 1.55  (1.33- 1.81) | <0.001* | 1.40  (1.20-1.64) | <0.001* | 1.37  (1.17-1.60) | <0.001* |
| Q4 | 1.82  (1.57- 2.12) | <0.001* | 1.44  (1.23-1.69) | <0.001* | 1.37  (1.16 -1.62) | 0.002* |
| TyG-WWI  (per 1 SD) | 1.16  (1.12-1.21) | <0.001* | 1.09  (1.04-1.15) | <0.001* | 1.08  (1.03-1.14) | 0.003* |
| TyG-WWI  Quartile |  |  |  |  |  |  |
| Q1 | Ref |  | Ref |  | Ref |  |
| Q2 | 1.49  (1.27-1.74) | <0.001* | 1.45  (1.24-1.69) | <0.001* | 1.44  (1.23-1.69) | <0.001* |
| Q3 | 1.53  (1.31-1.79) | <0.001* | 1.43  (1.22-1.68) | <0.001* | 1.41  (1.20-1.66) | <0.001* |
| Q4 | 1.88  (1.61-2.18) | <0.001* | 1.59  (1.35-1.88) | <0.001* | 1.52  (1.28-1.81) | <0.001* |
| TyG-CVAI  (per 1 SD) | 1.08  (1.05-1.10) | <0.001* | 1.26  (1.16-1.37) | 0.002* | 1.24  (1.13-1.35) | <0.001* |
| TyG-CVAI Quartile |  |  |  |  |  |  |
| Q1 | Ref |  | Ref |  | Ref |  |
| Q2 | 1.01  (0.86-1,18) | 0.930 | 1.00  (0.85-1.17) | 0.953 | 1.00  (0.83-1.16) | 0.979 |
| Q3 | 1.32  (1.13-1.53) | <0.001* | 1.24  (1.07 -1.46) | 0.005* | 1.25  (1.06-1.46) | 0.010* |
| Q4 | 1.82  (1.57-2.10) | <0.001* | 1.58  (1.35-1.85) | <0.001* | 1.56  (1.32-1.85) | 0.013* |

**Table S3 Cox Regression Analysis of TyG-Related Indices and New-Onset Hypertension Risk After Exclusion of Second-Wave Cases**

|  | Model I | P-valve | Model II | P-valve | Model III | P-valve |
| --- | --- | --- | --- | --- | --- | --- |
|  | HR (95% CI) |  | HR (95% CI) |  | HR (95% CI) |  |
| TyG-BRI  (per 1 SD) | 1.27  (1.19-1.37) | <0.001* | 1.27  (1.17-1.38) | <0.001* | 1.25  (1.15-1.36) | 0.001* |
| TyG-BRI  Quartile |  |  |  |  |  |  |
| Q1 | Ref |  | Ref |  | Ref |  |
| Q2 | 1.00  (0.80-1.26) | 0.971 | 1.02  (0.88-1.39) | 0.878 | 1.01  (0.81-1.27) | 0.928 |
| Q3 | 1.34  (1.09-1.66) | 0.006* | 1.36  (1.10-1.69) | 0.005* | 1.33  (1.07-1.66) | 0.011* |
| Q4 | 1.69  (1.38-2.06) | <0.001* | 1.72  (1.37-2.15) | <0.001* | 1.64  (1.30-2.07) | <0.001* |
| TyG-ABSI  (per 1 SD) | 1.27  (1.17-1.38) | <0.001* | 1.17  (1.08 -1.28) | <0.001* | 1.13  (1.04-1.24) | 0.004* |
| TyG-ABSI  Quartile |  |  |  |  |  |  |
| Q1 | Ref |  | Ref |  | Ref |  |
| Q2 | 1.32  (1.06- 1.64) | 0.014* | 1.26  (1.01-1.58) | 0.037* | 1.25  (1.00-1.56) | 0.051 |
| Q3 | 1.50  (1.21- 1.86) | <0.001* | 1.37  (1.10-1.70) | 0.004* | 1.31  (1.05-1.64) | 0.016* |
| Q4 | 1.84  (1.50- 2.27) | <0.001* | 1.51  (1.21-1.89) | <0.001* | 1.38  (1.10 -1.75) | 0.006* |
| TyG-WWI  (per 1 SD) | 1.28  (1.18-1.39) | <0.001* | 1.20  (1.10-1.31) | <0.001* | 1.16  (1.06-1.27) | 0.001* |
| TyG-WWI  Quartile |  |  |  |  |  |  |
| Q1 | Ref |  | Ref |  | Ref |  |
| Q2 | 1.29  (1.03-1.60) | 0.025* | 1.27  (1.01-1.58) | 0.037* | 1.25  (1.00-1.56) | 0.049* |
| Q3 | 1.38  (1.11-1.71) | 0.004* | 1.32  (1.06-1.65) | 0.014* | 1.28  (1.02-1.61) | 0.034* |
| Q4 | 1.93  (1.57-2.36) | <0.001* | 1.71  (1.37-2.14) | <0.001* | 1.59  (1.25-2.02) | <0.001* |
| TyG-CVAI  (per 1 SD) | 1.35  (1.26-1.44) | <0.001* | 1.30  (1.20-1.40) | <0.001* | 1.28  (1.18-1.40) | <0.001* |
| TyG-CVAI Quartile |  |  |  |  |  |  |
| Q1 | Ref |  | Ref |  | Ref |  |
| Q2 | 1.02  (0.81-1.28) | 0.872 | 1.00  (0.80-1.26) | 0.986 | 0.99  (0.79-1.25) | 0.964 |
| Q3 | 1.32  (1.06-1.64) | 0.011* | 1.28  (1.03 -1.60) | 0.029* | 1.26  (1.01-1.58) | 0.041* |
| Q4 | 2.03  (1.66-2.48) | <0.001* | 1.85  (1.48-2.30) | <0.001* | 1.79  (1.42-2.26) | <0.001* |

**Table S4 E-value for the TyG-related index in the fully adjusted model**

|  | **E-value-point** | **E-value-lowerCI** |
| --- | --- | --- |
| **TyG-BRI** | 1.71 | 1.54 |
| **TyG-ABSI** | 1.4 | 1.21 |
| **TyG-WWI** | 1.49 | 1.28 |
| **TyG-CVAI** | 1.71 | 1.54 |
